# A Novel Strategy in Electromagnetic Wave Absorbing and Shielding Materials Design: Multi‐Responsive Field Effect

**DOI:** 10.1002/smsc.202100077

**Published:** 2021-11-27

**Authors:** Yue Zhao, Lele Hao, Xindan Zhang, Shujuan Tan, Haohang Li, Jing Zheng, Guangbin Ji

**Affiliations:** ^1^ College of Materials Science and Technology Nanjing University of Aeronautics and Astronautics Nanjing 211100 P. R. China; ^2^ Department of Chemistry and Materials Science College of Science Nanjing Forestry University Nanjing 210037 P. R. China

**Keywords:** electromagnetic wave absorbers, field effects, multi‐responsive properties of electromagnetic wave absorbing and shielding materials

## Abstract

The electromagnetic (EM) devices have been widely used in communication, electrical engineering, and medical care. However, EM device is a double‐edged sword for its convenience is followed by signal pollution and radiation. Electromagnetic interference (EMI) combat has brought lots of attention to researchers in this field. Researchers have made great efforts in developing electromagnetic wave absorbing and shielding (EMAS) materials to reduce EM wave power density to solve the above problem. However, the great majority of reported EMAS materials are powders and coatings, which possess merely EMAS property. Modern practical application has abundant multiple scenes, including high temperature, intense light, water flow, etc. Under the circumstances, EMAS materials should be functionalized with outstanding tunable absorption bands. Based on field theory in physics, multiexternal fields‐responsive materials are an effective method to deal with the above urgently unsolved problem. Thus, herein, different external field‐responsive materials, including temperature, light, space–time, electrical, wind, density, and flow fields, are focused on. Various action mechanisms, materials synthesis methods, and different macrostructures are summarized in detail. Meanwhile, the developing trends of novel external field‐responsive materials are also discussed in order. Finally, the challenges of designing new type of EMAS devices are mentioned.

## Graphical abstract

The current multifunctional electromagnetic wave absorbing and shielding (EMAS) materials are reviewed. An external field‐responsive strategy is proposed to enrich application scenes, including temperature field, light field, space–time field, electrical field, wind field, density field, and flow field. Action mechanisms and EMAS performance improvements are discussed and concluded.

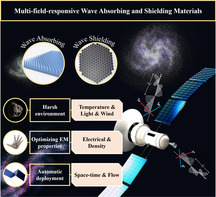

## 1 Introduction

With the rapid development of 5th Generation Mobile Communication Technology (5G), late‐model electromagnetic wave absorbing and shielding (EMAS) materials have proven to be the research emphasis of electromagnetic (EM) wave shelter. Great efforts have been made to optimize EMAS functional materials for diverse EM wavebands.^[^ ^1^ ^]^ Each range of EM wave bands has a specific application in communication, electrical engineering, or information transmission.^[^ ^2^ ^]^ For instance, ultrahigh frequency (UHF) EM wave of 0.3 < *f* < 3 GHz, also known as P/L/S or B/C/D/E band, is applied to television radar air navigation and mobile communication as well as microwave relay systems; the superhigh frequency (SHF) EM wave of 3 < *f* < 30 GHz, including S/C/X/Ku/K/Ka‐band or F/G/H/I/J/K band, is customarily adopted in digital telecommunication and satellite communication as well as waveguide communication. Moreover, EM waves in other frequency ranges, like terahertz wave and gamma ray, have been widely used in label‐free DNA detection, high‐temperature superconducting material research, radiographic testing, and even oncotherapy. In these cases, each specific application scenario requires specific EM waves in a specific frequency range, illustrating that EM wave clutters should be absorbed or otherwise shielded. Under these circumstances, developing novel and practical EMAS materials may meet the demands of EM wave science, especially in multifunctional fields.

Traditional EMAS materials consist of resistance absorber,^[^ ^3^ ^]^ dielectric absorber,^[^ ^4^ ^]^ magnetic medium absorber,^[^ ^5^ ^]^ and composites based on monomaterials as mentioned earlier,^[^ ^6^ ^]^ including CuS,^[^ ^7^ ^]^ MoSe_2_,^[^ ^8^ ^]^ ferrites and alloys (Co_*x*_Fe_3−*x*_O_4_@C),^[^ ^9^ ^]^ modified carbons (hollow carbon spheres),^[^ ^10^ ^]^ etc. In the past decade, 2D transition metal carbides or nitrides, or MXenes, have been prefabricated and naturally applied to EMAS materials synthesis due to their outstanding EM wave loss capacity.^[^ ^11^ ^]^ Many scholars have devoted themselves to MXene materials and structure design. The electrical conductivity of MXenes can reach 10^3^ S cm^−1^, leading to extraordinary EM wave shielding property.^[^ ^12^ ^]^ Many reported MXene absorbers are monolayers,^[^ ^13^ ^]^ whereas macrostructure design is introduced. Thus, macrodevices, like hollow MXene sphere foam,^[^ ^14^ ^]^ are synthesized. It is therefore expanded that the ecosystem of EMAS materials’ formation reaches an unprecedented abundant level. Metamaterials refer to composites with artificially designed structures and present extraordinary physical properties that natural materials do not possess. Heterostructures, like copper/graphene film,^[^ ^3^ ^]^ flexible ZnO/carbon cloth,^[^ ^15^ ^]^ and hierarchical metamaterials,^[^ ^16^ ^]^ have become promising potential EM wave absorbers or shielding devices. Several sorts of research have been conducted about the metacarbonic systems, including graphene^[^ ^17^ ^]^ and multiwalled carbon nanotubes (MWCNTs).^[^ ^18^ ^]^ In virtue of its unique micro–macrostructure, the metamaterial can realize exceptional response ability, including ultralow frequency absorption,^[^ ^19^ ^]^ multiband absorption,^[^ ^20^ ^]^ and terahertz harvester.^[^ ^21^ ^]^ The volume of publications focusing on materials synthesis and structure design is rising year by year. Yet, current research trends do not satisfy practical demands well enough. Productions are often used in several different external environments, requiring the EMAS materials as multi‐responsive mediums to diverse fields.

**Figure 1:**
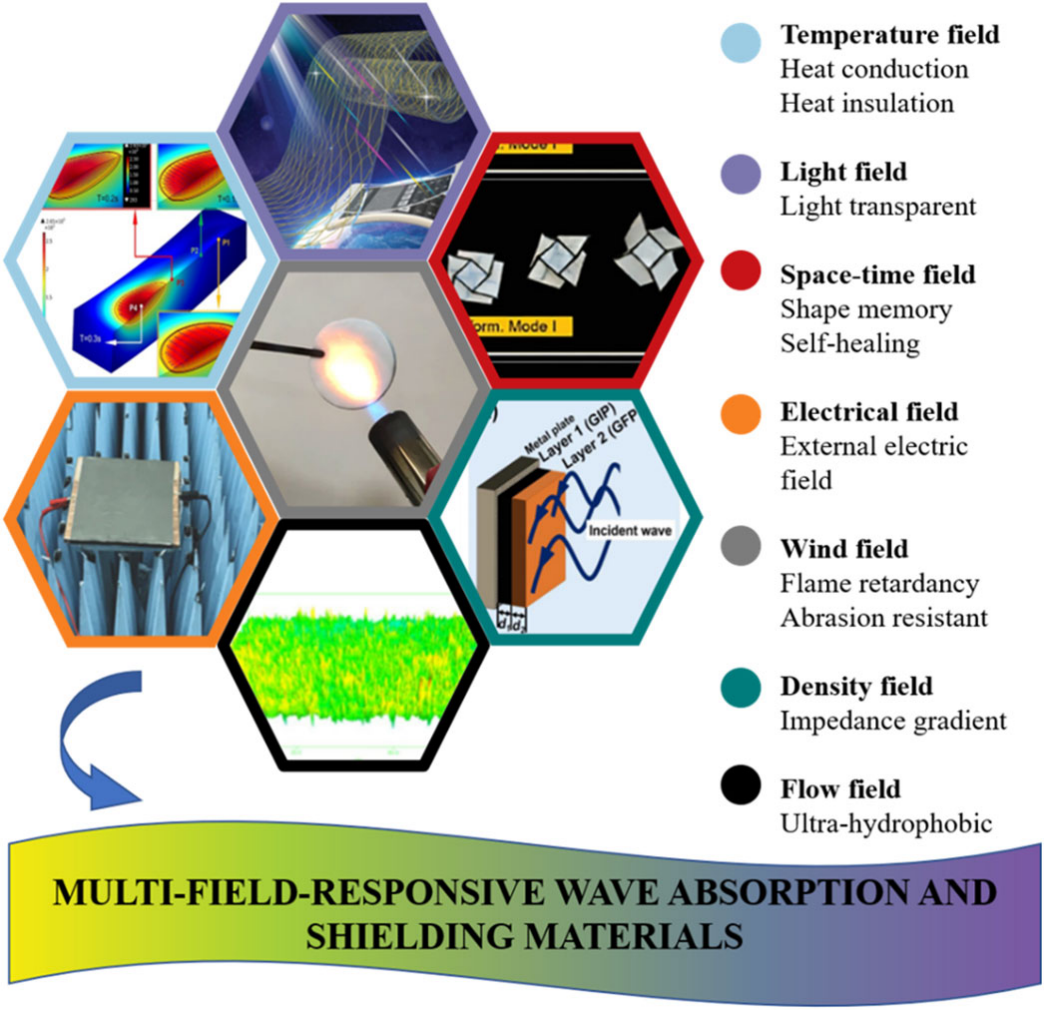
The schematic illustrations of typical multifield‐responsive wave absorption and shielding materials. Temperature field: Reproduced with permission.^[^ ^112^ ^]^ Copyright 2021, Elsevier. Light field: Reproduced with permission.^[^ ^113^ ^]^ Copyright 2017, Royal Society of Chemistry. Space–time field: Reproduced with permission.^[^ ^58^ ^]^ Copyright 2020, Wiley‐VCH. Electrical field: Reproduced with permission.^[^ ^80^ ^]^ Copyright 2018, Wiley‐VCH. Wind field: Reproduced with permission.^[^ ^84^ ^]^ Copyright 2021, American Chemical Society. Density field: Reproduced with permission.^[^ ^100^ ^]^ Copyright 2020, Elsevier. Flow field: Reproduced with permission.^[^ ^105^ ^]^ Copyright 2020, Elsevier.

**Figure 2:**
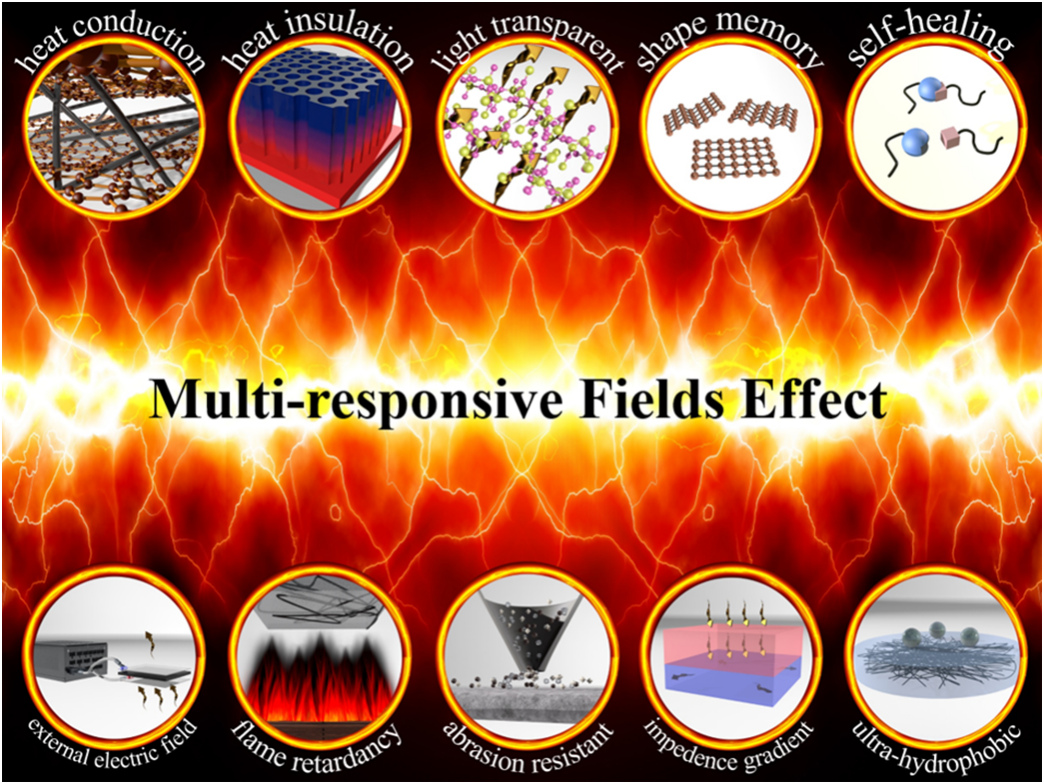
The schematic illustration of mechanism about multi‐responsive field effect of different external fields.

To multiply and enlarge the application scenarios, this work innovatively contacts the multifunctionality‐to‐multifield response. Each feature can be linked with a specific field. Though the field theory, this review summarizes EMAS materials’ field‐responsive abilities, including temperature field, light field, space–time field, electrical field, wind field, density field, and flow field. **Figure** **1** shows the common field theories to which EMAS materials are sensitive. **Figure** **2** shows the basic mechanism of seven different external field effects. First, the diverse external fields correlated with EMAS functional properties are discussed. Second, different action mechanisms affected by physical fields are proposed. Third, material system, EMAS property, physical characteristics under external field, and application situations are presented. Finally, current research trends and challenges in materials synthesis, applied physical field mechanism development, and EMAS production are discussed.

## 2 EMAS Materials Mechanism and Theory

EMAS materials can absorb incident EM waves and make the energy conversion of forwarding EM waves into thermal energy and other energy formation or reflect incident wave and reduce transmitted beam. EMAS properties are both calculated by a vector network analyzer (VNA) through the two ports *S* (scattering) parameters.^[^ ^22^ ^]^ Most EM wave functional materials systems are applicable in the frequency range of 2.0–18.0 GHz. Coaxial‐line and waveguide methods are two commonly used measurement methods in electromagnetic spectroscopy. Monolayer metal backplane model is proposed to simulate actual coating or film.^[^ ^23^ ^]^

### 2.1 EM Wave Absorbing Materials

The EM wave absorber emphasizes low‐reflection wave beam amplitude, which means that it has to absorb incident EM waves as much as possible. Modern electronic devices present new demands on high‐value practical applications that a great absorber needs a strong absorption peak with a broadband frequency range and is compatible with low density and thin thickness. There are two customarily adopted parameters to evaluate the absorption ability of EM absorbing materials. One variable is reflection loss (RL), determined by amplitudes of reflection wave, transmission wave, and incident wave, and uses decibel (dB) as a unit. RL is calculated by Equation (1) and is a negative value determined by a logarithmic function. To quantify the absorption property better, RL should be less than −10 dB through artificial regulation in most cases, which means 90% of the corresponding EM wave absorbed.

(1) $$\text{RL}=20\;\log_{10}|\frac{Z_{\text{in}}-Z_{0}}{Z_{\text{in}}+Z_{0}}|$$

*Z* in Equation (1) refers to impedance determined by relative EM parameters, where *Z*_in_ is normalized input impedance of the air‐coating interface, and free space impedance, *Z*_0_, is 1. As *Z* is normalized relative impedance, *Z*_in_ and *Z*_0_ are both dimensionless.^[^ ^24^ ^]^ Based on transmission theory, *Z*_in_ is related to EM parameters in Equation (2), relative complex permittivity *ε*_r_ (*ε*_r_ = *ε*′–*jε*″), and relative complex permeability *μ*_r_ (*μ*_r_ = *μ*′–*jμ*″).^[^ ^25^ ^]^

(2) $$Z_{\text{in}}=\sqrt{\mu_{r}/\varepsilon_{r}}\tanh(j\sqrt{\mu_{r}\varepsilon_{r}}2\pi d/\lambda )$$

Here, *d* is the thickness of the coating, and *λ* is the wavelength of the incident EM wave. The EM parameters can be obtained through *S* parameters, including *S*_11_, *S*_12_, *S*_21_, and *S*_22_, where 1 and 2 refer to port order.

### 2.2 EM Wave Shielding Materials

Shielding material concentrates on heterology signal launcher and receiver, which means that the coating or film combined with shielding property can have a high reflectance, distinguished from absorbing material. Former researchers propose the shielding effectiveness (SE) to characterize EM SE, and it can be expressed by Equation (3).

(3) $$\text{SE}={\text{SE}}_{R}+{\text{ SE}}_{A}+{\text{SE}}_{M}$$

Here, *R*, *A*, and *M* are reflection, absorption, and multireflection, respectively. All of them are expressed in dB. For monolayer film or coating, SE_M_ is usually too small to take into consideration. Thus, SE is mainly determined by reflection and absorption. SE_R_ and SE_A_ can be calculated by Equation (4) and (5) through *S* parameters.^[^ ^26^ ^]^

(4) $${\text{SE}}_{R}=-10\;\log_{10}(1-{\left|S_{11}\right|}^{2})$$

(5) $${\text{SE}}_{A}=-10\;\log_{10}[{\left|S_{12}\right|}^{2}/(1-{\left|S_{11}\right|}^{2})]$$

In addition, shielding property is usually calculated by specific SE (SSE) (SE divided by density) and further characterized by the parameter of SSE/*t* (SSE divided by thickness).

Customarily, SE should be at least 20 dB to ensure minimum transmission of EM waves. Generally speaking, an absorbing material has the feature of low reflection; a shielding material, in contrast, must possess low transmission.

## 3 Temperature Field

The field is a mapping of one vector to another vector or number, mathematically, indicating that it consists of vector quantity fields and scalar quantity fields. In this case, the temperature field is a representative scalar field. To ensure EMAS materials are applicable to mutable temperature environments, temperature field theory is proposed. While EMAS materials are working, EM wave energy is transferred into thermal energy mostly, and thus heat conduction or heat insulation is needed to minimize the heat and maintain electronic devices in good condition. Extensive research productions have focused on combining heat conduction or heat insulation with microwave absorption or shielding.

### 3.1 Heat Conduction

A proper heat conductor requires high thermal conductivity in W m^−1^ K^−1^. Metal, alloy, and CNTs are most commonly applied in this domain, which are also highly electrically conductive leading to high SE. To smoothly conduct thermal energy, porous structures or foams should be avoided, which make the energy transition path complex and zigzag. Devices, like smartphones, produce massive thermal energy, in which case EMAS materials, like patches, should be temperature field responsive and compatible with high heat conductivity.

**Figure 3:**
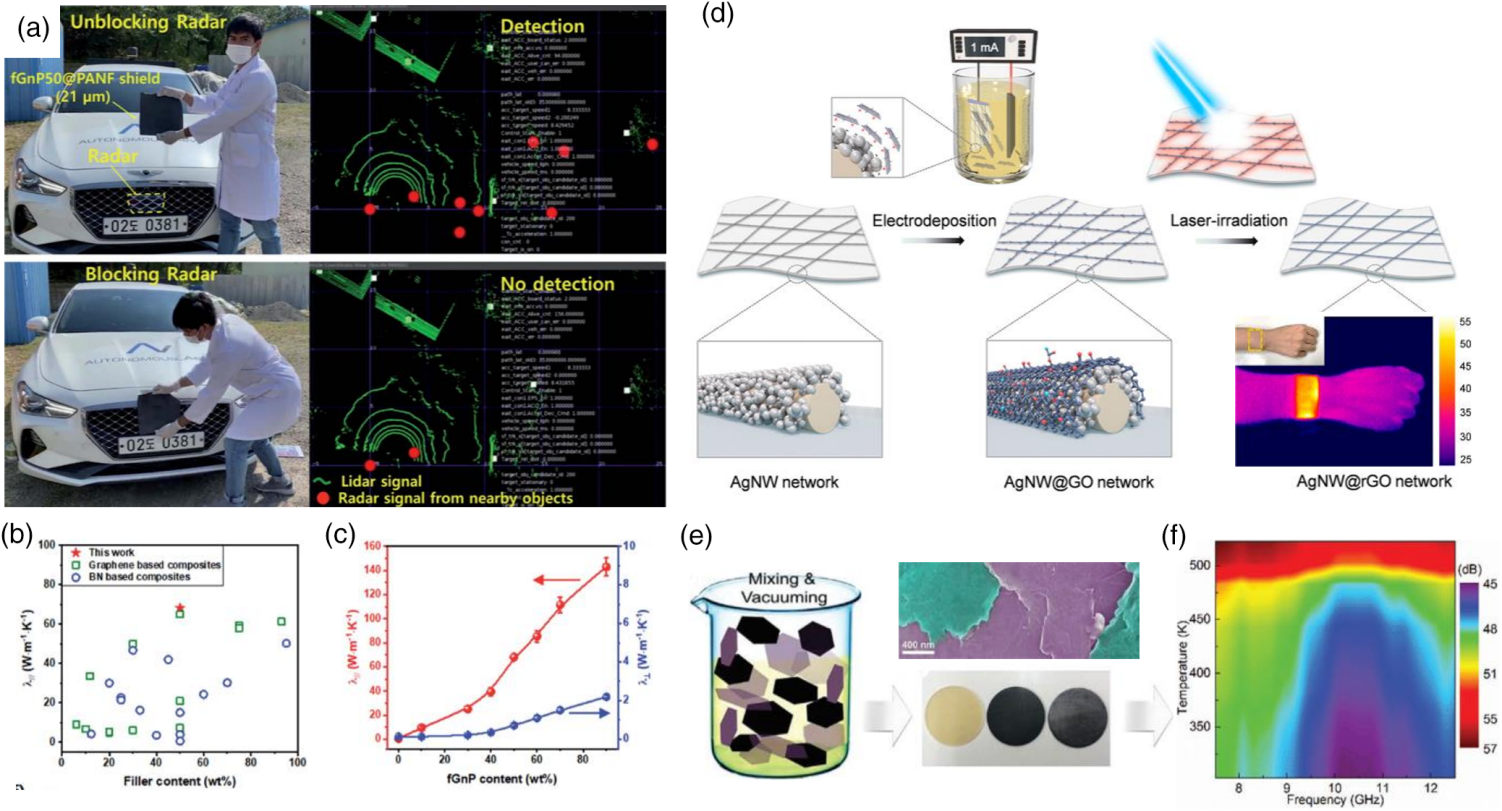
a) Radar signals before and after being blocked by shielding paper. b) Summary of the thermal conductivity of different nanocomposites. c) Thermal conductivity of as‐prepared composites. a–c) Reproduced with permission.^[^ ^27^ ^]^ Copyright 2021, Royal Society of Chemistry. d) Schematic illustration of AgNW@rGO. Reproduced with permission.^[^ ^28^ ^]^ Copyright 2020, American Chemical Society. e) Process flow for the sample preparation. f) EM wave shielding efficiency as a function of temperature. e–f) Reproduced with permission.^[^ ^29^ ^]^ Copyright 2020, Wiley‐VCH.

As shown in **Figure** **3**a–c, graphene nanoplatelet crosslinked aramid nanofiber papers were fabricated via a self‐assembly approach.^[^ ^27^ ^]^ This paper was cut into squares and put before a car detective radar to verify its shielding property. After being covered by shielding papers, radar signals from nearby objects could no longer be detected from vehicle radar. As an ultrathin graphene‐based paper, it showed a superb thermal conductivity of 68.2 W m^−1^ K^−1^, overmatching metal productions as Figure 3b shows. This paper contributes to mass‐scale commercial production for graphene‐based shielding materials. Graphene is a typical 2D carbon material with high electrical and thermal conductivity. Based on the synergistic effect of 1D nanomaterials and 2D carbon materials, Yang et al. designed a reduced graphene oxide (rGO) composite wrapped by a silver nanowire (NW) as a conductive network, as shown in Figure 3d.^[^ ^28^ ^]^ These 1D−2D composites exhibited high Joule heating temperature and could conduct body heat through infrared thermal imaging. The network of the 1D−2D system improves the electron transport capacity, and rGO reduces the oxygen and water molecules within composites, leading to excellent heat conducting ability, as reported. Barani et al. made further efforts to graphene composites as EM wave shielding material with proper thermal performance.^[^ ^29^ ^]^ An epoxy few‐layer graphene coating was described with SE = 65 dB at 1 mm thickness, revealing thermal conductivity of 11.2 W m^−1^ K^−1^ (Figure 3e,f). The samples were prepared through two‐step methods of mixing and vacuuming graphene flakes with epoxy. Affected by the thermal management ability, this composite shows excellent SE during temperature change. It is necessary for next‐generation high‐temperature electronic devices.

Considering practical application, it is required that EMAS materials have multifunctional properties. To construct multifunctional temperature field‐responsive EM shielding material, Li et al. designed mechanically robust, thermally conductive graphene composites.^[^ ^30^ ^]^ The thermal conductivity of graphene/nickel (RGN) paper reached 247 W m^−1^ K^−1^. Due to nickel network, RGN paper was fire resistant after burning in fire for 30 s. The RGN paper reached 55 dB of SE at 0.12 mm in X‐band. The fabrication of RGN paper paves the way for spacecraft surface coating production. Graphene is a primarily used monomer in shielding area. Still, 1D material, like CNT, is also very auspicious. Jia et al. built a thick film consisting of graphene and CNT, with a thermal dissipation feature.^[^ ^31^ ^]^ It was prepared through two‐step chemical synthesis, hydrogen iodide reduction and high‐temperature reduction. By orientation of CNT, heat radiation could dissipate directionally, which was vital for short‐term thermal dissipation. After being heated to 97.1 °C, graphene/CNT film cooled to 36.6 °C in 12 s. The corresponding thermal conductivity was 933.37 W m^−1^ K^−1^ with an SE of 75 dB. Apart from graphene, 1D CNT combined with metallic oxide is also a strategy to facilitate EM absorption ability with an external temperature field. Zhou et al. developed CNT@NiO/rubber composites with great impedance matching.^[^ ^32^ ^]^ The RL was remarkably improved to −43.6 dB at 17.5 GHz with a thickness of 1.3 mm due to interfacial polarization, dipolar polarization, and conductive loss. This flexible material exhibited a heat dissipation property of cooling down to room temperature after being heated to 84.4 °C in 3 min for NiO serving as an intermediate between CNT and rubber. This result indicates that both graphene and CNT have excellent potential in thermal management. Similarly, MWCNTs/waterborne polyurethane (WPU) composite film was reported by Liu et al. via in situ polymerization.^[^ ^33^ ^]^ The as‐prepared film exhibited an excellent SE of 65.3 dB at a low‐frequency range, 110 MHz, with an ultrathin thickness of 80 μm, which could block 99.9999% of the incident wave. It could be demonstrated that MWCNTs twined by WPU granules provided a plentiful conductive path from the microzone composition analysis and thermal–electrical performance test. Furthermore, the composites exhibited a stable EM shielding property after bending 1000 times.

In addition, MXene is another 2D material with superior conductivity and EM wave activity. MXene has caught much attention in the relative research domain due to its unique layered graphene‐like nanostructure. Wang et al. manufactured a MXene textile using poly (ethylene terephthalate) (PET) textile through dip coating, and Ti_3_C_2_T_*x*_ was selected as the main ingredient.^[^ ^34^ ^]^ The multilayer MXene structure showed significant electromagnetic interference (EMI) SE of 90 dB, along with excellent Joule heating performance at a driving voltage of 1.5 V. The as‐prepared MXene textile could be heated to 58 °C within 2 min. MXene can easily get oxidized in open air, even though it has ultrahigh conductivity and shielding performance. To lengthen the life span of MXene material, Lu et al. prepared a 2D MXene material based on rubber to isolate oxygen.^[^ ^35^ ^]^ Ethylene propylene diene rubber (EPDM) was selected as a basement. With the MXene content at 6 wt%, the composites exhibited 1.57 W m^−1^ K^−1^ thermal conductivity. As for EMI SE of MXene/rubber, it reached 52 dB in 12.4–18 GHz at 0.3 mm. It can be concluded that even with ultralow filling levels, MXene is still not to be ignored for creating next‐generation electrons.

Papers focused on graphene, CNT, and MXene mainly concentrate on microcomponent regulation, whereas the structural design is another significant way to improve thermal and EMAS ability. Chen et al. were inspired by kirigami, which is a traditional Japanese paper cutting artistic form, and designed a Ti_3_C_2_T_*x*_ MXene film as a pressure sensor.^[^ ^36^ ^]^ It was built into stretchable electronics and showed an EMI SE shielding of 30 dB. In addition, this kind of MXene film was highly stable even if it was stretched to 150% of original length, and its thermal conductive ability was verified through infrared camera images. The Kirigami‐inspired film could be heated to 140 °C in 180 s. Not only is microstructure suitable for functional material, but macrodesign is also a practical method to optimize EMI SE. It was reported that rGO–graphene foam could be synthesized by the bubble template method.^[^ ^37^ ^]^ The foam was created by a ball‐milling machine and rotating beaker; during this procedure, macrobubble came into being at the center and surface of the rGO–graphene foam. A 2 mm‐thick foam possessed an 86 dB SE value with a conductivity of 3 W m^−1^ K^−1^. The pore structure created multireflection tunnels for incident EM waves, thus leading to a great EMI SE. To further improve thermal management function, the researcher used thermal isolation as the topic, followed by new material systems and procedures.

### 3.2 Heat Insulation

A large number of EMAS devices or coatings are used in hot environments; in this circumstance, heat insulation is as vital as heat conduction in an external temperature field. Therefore, researchers have developed various structures and components to fulfill heat insulation purposes. Heat insulation requires low thermal conductivity compared with heat conduction, which can be realized through porous materials or ceramics. However, porous structure leads to the low electrical property. Thus, how to achieve heat insulation and EMAS becomes the real challenge.

**Figure 4:**
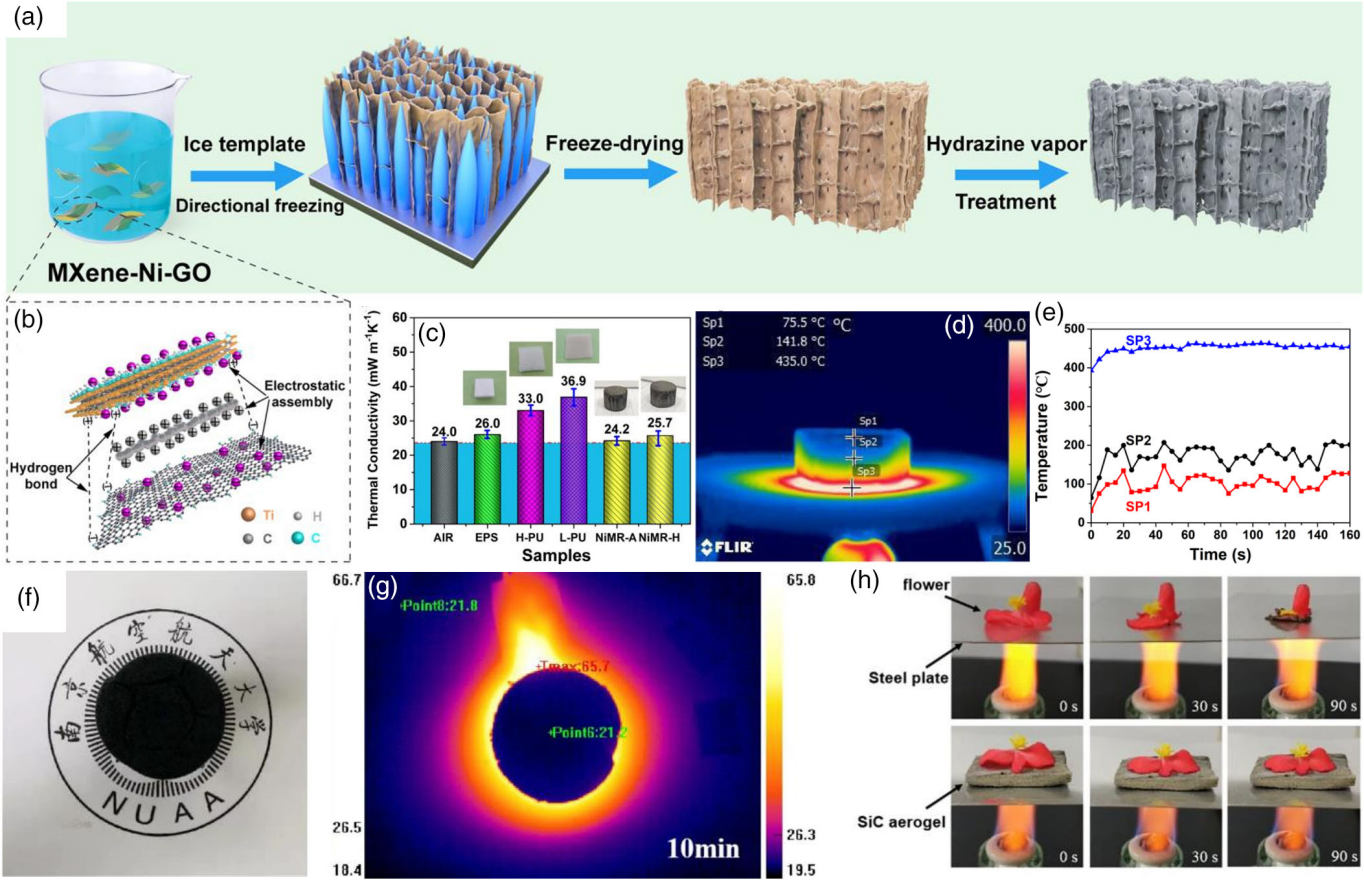
a) Schematic illustration of fabricating aerogel. b) Electrostatic and bonding effect between MXene, Ni, and GO. c) Thermal conductivities of different aerogels. d) Infrared thermal image of aerogel while heating. e) Temperature–time curves. a–e) Reproduced with permission.^[^ ^38^ ^]^ Copyright 2021, American Chemical Society. f) Physical drawing of sample. g) Thermal infrared image. f–g) Reproduced with permission.^[^ ^39^ ^]^ Copyright 2020, Elsevier. h) Heat‐resistant properties of SiC aerogels. Reproduced with permission.^[^ ^40^ ^]^ Copyright 2019, Elsevier.

**Figure** **4**a–e shows a strategy to fabricate MXene–Ni–GO aerogel with the multifunctional property.^[^ ^38^ ^]^ Liang et al. used a freeze dryer and vapor reduction process to build an aerogel structure to realize heat isolation. The minimal RL value of the as‐prepared system was −75.2 dB with an absorption band of 7.3 GHz. By forming microcell units and dielectric and magnetic interfaces, multipolarization and impedance matching were significantly improved. From Figure 4d, it was clear that MXene–Ni–GO aerogel perfectly isolated thermal energy from heating source to air. In this way, by constructing aerogels or porous structures, heat isolation can be realized quickly, attracting a lot of attention. Figure 4f,g shows that a cellulose–chitosan/polyaniline (PANI) aerogel was constructed and compatible with heat isolation.^[^ ^39^ ^]^ When the heating plate was 65 °C, the core area of the aerogel was maintained at 18 °C. More than that, cellulose–chitosan/PANI composites also possessed fabulous EM performance with −54.76 dB as most substantial absorption and 6.04 GHz broadband. The EM performance can be improved from the filler ratio of the absorber, while cellulose provides a porous structure.

Biomass materials are widely used in the chemical industry, owing to the low cost and efficient manufacturing process. Liang et al. got inspired by eggplant and developed a SiC aerogel with the outstanding property.^[^ ^40^ ^]^ Eggplants were dried and cut into 1 cm blocks, followed by pyrolyzing and carbothermal reduction. Figure 4h shows the thermal isolation ability of SiC aerogel after being heated in 90 s. Also, 1D SiC nanowires were obtained through the procedure, contributing an interconnected network for EM absorbing. The density of SiC gels was 0.076 g cm^−3^, much lower than most absorbing materials. For reclamation and recycling, Gu et al. used shaddock peel as raw material to fabricate biomass carbon aerogel.^[^ ^41^ ^]^ Shaddock peel was frozen dried and prepared by pyrolysis through two‐step methods. The shaddock peel carbon gels possessed a minimum RL of −29.5 dB with 5.8 GHz bandwidth, and they had a very low heating rate while being put on a hot platform. The 3D porous conducting network provides a wide range for electron migration, leading to massive conduction loss. In a similar research result, Zhao et al. chose pomelo peel as an ingredient.^[^ ^42^ ^]^ Using different procedures, pomelo peel could be transferred into various forms; a single H_2_O_2_ treatment sculptured it into a brick‐like porous 3D material, and single HAc treatment could peel pomelo off into a thin carbon nanosheet with a porous structure. By combining the mentioned two methods, graphene‐like porous carbon was successfully synthesized with −56.4 dB minimum RL and 6.4 GHz absorption bandwidth. Its heat isolation property was better than carbon foam (CF) and other commercial production.

Apart from biomass‐inspired materials, an organic–inorganic system is also applied to optimize EMAS and thermally insulating. Li et al. used organic fibers and membranes as crosslink mediums to form a 3D network aerogel.^[^ ^43^ ^]^ Fe_3_O_4_ magnetic particles were dispersed into aerogel. After being freeze dried, the composites were crosslinked closely by polymerization. Through infrared images, as‐prepared aerogel was confirmed to have a multifunctional application. It cooled the heating platform down for at least 30 °C and exhibited a −59.85 dB absorption ability at the thickness of 1.5 mm, due to organic–inorganic materials synergistic effect. To further review the organic–inorganic system, metal–organic frameworks (MOFs) are introduced. Gu et al. developed a hybrid foam grown out of Co‐based MOFs.^[^ ^44^ ^]^ Remarkably, the hybrid foam could reach a minimum RL value of −59.82 dB at a thickness of 2.3 mm. The unique MOF structure brought up dipole polarization and high conductive loss. The porous structure was able to convert thermal energy into air. This work combined MOFs with aerogel structures, inspiring the preparation of macroscale EMAS multifunctional materials. To better isolate heat energy, a directional controlled growth method was applied.^[^ ^45^ ^]^ Directional controlled growth was realized by ultraquick freezing using liquid nitrogen. Ice crystals grew inside the suspension and squeezed MXene and CNT nanoparticles; thus, directional MXene/CNT aerogel was fabricated. EMI SE was tested as 51 dB with an ultralow filling ratio of 0.3%. In addition, MXene/CNT aerogel reduced by nearly 65 °C temperature while put on the heating platform. Benefiting from controlled directional growth, aerogel was compressible, broadening its application situation. TiO_2_ also has the potential as a thermal radiation shielding material. Yu et al. reported an in situ‐grown TiO_2_ nanorod aerogel.^[^ ^46^ ^]^ The thermal conductivity of TiO_2_ nanorod aerogel was 0.071 W m^−1^ K^−1^. When it was heated to 1200 °C on the one side, the other side of the nanorod aerogel was only 100 °C, causing its superior heat isolation property. Cavities, constructed by TiO_2_ fibers, played an essential role in heat insolation while heat source was applied. TiO_2_ nanorod aerogel could stay stabilized even over 1200 °C. The biomimetic structure is a significant topic in discussion in recent years. Inspired by hairy skin, Choe et al. developed a micro‐/nanoporous material via vapor‐induced method to separate different phases.^[^ ^47^ ^]^ It could isolate at least 20 °C while heating and reduce heat energy over 61.4% with the concentration of polylactic acid as variable. The hairy pillar was beneficial to dissipation of heat, making hot objects unseen under the infrared camera. The outstanding thermal shielding property of this micro‐/nanomaterial extended the application in the military or aerospace area.

In general, after being applied in the external temperature field, EMAS materials can possess heat management ability in two ways. One effective method is heat conduction. To realize high conductivity, carbon materials, like graphene and CNT, along with MXenes, are effective constituents. Films and papers are often used macrostructures to conduct heat energy. Another commonly reported method is heat isolation. Porous structures, like foam and aerogel, can realize outstanding thermal shielding. However, many temperature field‐responsive EMAS materials face restrictions in oxidative resistance and mechanical durability due to weak interaction, which should be the following research centrality in the future. In addition, new types of temperature field‐responsive EMAS materials should be developed.

## 4 Light Field

The light field is a vector field, defined by Faraday. With the development of transparent electrical equipment, like transparent television, EM optical window, photochromic coating, and transparent screen, a mature transparent or light field‐responsive EMAS material is desperately needed. To construct a transparent EMAS material, transparent polymer and high‐level conductivity components for realizing excellent EMAS property with low filling ratios attract attention. Likewise, Ag‐based and MXenes‐based composites are hot topics nowadays. Furthermore, the flexible device is also a material system under consideration due to the fact that most of the transparent equipment is flexible.

**Figure 5:**
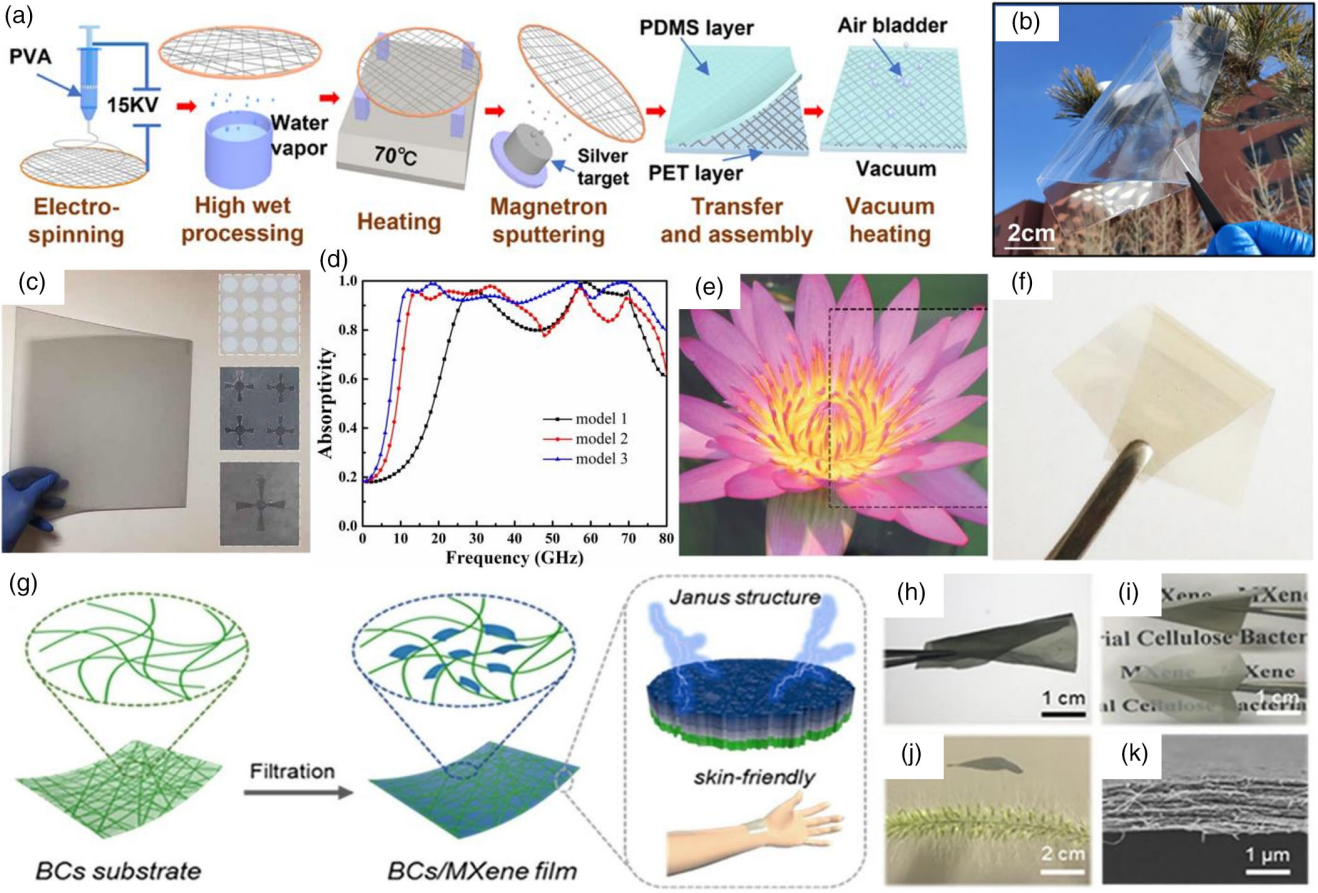
a) Fundamental fabrication process flow for flexible and transparent AgNT networks. b) Image of a folded AgNT network in the natural environment. a–b) Reproduced with permission.^[^ ^48^ ^]^ Copyright 2021, American Chemical Society. c) Fabricated metamaterial prototype. d) Contribution from different elements to absorbance. c–d) Reproduced with permission.^[^ ^49^ ^]^ Copyright 2021, IOP Publishing, Ltd. e–f) Digital photograph of the PET/Ag NW/PMMA flexible transparent conductive films. Reproduced with permission.^[^ ^50^ ^]^ Copyright 2021, Royal Society of Chemistry. g) Schematic of the BCs/MXene film preparation process. h–j) Optical images show the BCs/MXene films. k) The cross‐section scanning electron microscopy (SEM) image shows the lamellar structure. g–k) Reproduced with permission.^[^ ^51^ ^]^ Copyright 2020, Elsevier.

AgNT‐based EMI shielding film was developed, as shown in **Figure** **5**a,b.^[^ ^48^ ^]^ Through the electrospinning method, uniform AgNT was wrapped between PET and poly (dimethylsiloxane) (PDMS) layers for protection in case of oxidation. The unique sandwich structure ensured that the film achieved high transparency with a transmittance of 90% in the visible light frequency range between 400 and 800 nm. This transparent film could realize 35 dB EMI SE at the low thickness of 1 mm, which could block a 4G cellphone signal. Indium tin oxide (ITO) is also a customarily used transparent absorber. Deng et al. reported an ITO‐based metamaterial absorber (MMA), which combined EM absorbing and light transparent properties as well, as shown in Figure 5c,d.^[^ ^49^ ^]^ Numerical simulation analysis was applied to construct architectural details of metamaterial, and from the simulation outcomes, more than 90% of EM was absorbed between 10 and 75.5 GHz. Optical transparency was measured at the wavelength range of 400–800 nm. The multilayered structure was confirmed to have high light transmissivity close to 60%. Just like carbon materials’ multiforms, silver was designed into various microformations, including nanowires. Ag NW‐based composites were applied as one of the coatings to build a sandwich‐like transparent film in Figure 5e,f.^[^ ^50^ ^]^ Polymethyl methacrylate (PMMA) and PET were used as upper and lower coatings of sandwich‐like material, respectively. The EMI SE was 21.3 dB in 8–12 GHz with a density of 90 mg m^−2^. Protected by two polymer layers, Ag NWs exhibited excellent stability after aging for 600 h; the EMI SE values remained almost unchanged afterward.

Ma et al. adopted another material system as an absorber.^[^ ^51^ ^]^ Bacterial celluloses/MXene composites were fabricated into Janus structure, which was named after an ancient god with two faces in Roman mythology, indicating that this structure had different components from two directions. This creative film exhibited a specific SE (divided by thickness) of 69455.2 dB cm^2^ g^−1^ at 1.732 μm. Janus structure was constructed by vacuum filtration method with an ultralow MXene concentration of 0.005 mg mL^−1^. Its unique components and structure shine the path of a wearable EMAS film for intelligent devices. Copper and silver are both high‐conductivity metal elements, leading to excellent EMI SE. Similarly, the Cu NWs‐based sandwich conducting network was a promising EM shielding candidate.^[^ ^52^ ^]^ First, PET and Cu NWs were shaped by vacuum filtration, and polyethersulfone (PES) was grown onto the composites by spin coating. Cu NWs‐based sandwich shielding material was constructed with 22 dB SE value and 73% visible light transmittance through the simple two‐step methods. It also exhibited excellent chemical stability in the air within 45 days or in acid solution, increasing operating life as wearable electronics. To further improve the EMI SE property, Voronin et al. combined Cu and Ag on a cracked template as EM shielding coating.^[^ ^53^ ^]^ Cu–Ag and Ni–Ag meshes were synthesized by sputtering sequentially. Cu–Ag mesh increased EMI SE to 43.7 dB with light transparency of 82.2% and sheet resistance of 0.25 Ω sq^−1^. Alongside excellent light transparency, Cu–Ag and Ni–Ag meshes possessed high‐frequency shielding ability in 18–26.5 GHz with a thickness of only 0.95 μm. The EMI SE and sheet resistance could be controlled by treatment time.

MMA is constructed by artificial structures with subwavelength size, having unusual EMAS properties. Due to its unique EM parameters, metamaterial becomes the hot topic of relative researchers. Zhang et al. used ITO, PET, and PMMA to design a water‐based MMA, which could absorb a 5.8–16.2 GHz range of EM wave frequency.^[^ ^54^ ^]^ Owing to the transparent basements, MMA exhibited a 70.18% visible light transmittance between 400 and 800 nm. The introduction of the water layer effectively increased optical transmittance from 56.05% to 70.18%, and the most proper water layer height was 1.2 mm. More than that, as‐prepared MMA exhibited excellent absorptivity in wide range incident angle from 0° to 45°. Xu et al. also applied ITO as light field‐responsive MMA.^[^ ^55^ ^]^ It was reported that this MMA was composed of ITO and glass substrates with low RL, including quartz glass and soda‐lime glass. ITO‐based MMA could absorb EM waves between 8 and 20 GHz with a wide effective incident angle from 0° to 60° and 80% visible light transparency with high sensitivity to polarization. The EM absorption ability was studied through simulation and experiment, which demonstrated outstanding EM absorption ability. Dual‐band or multiband absorber has been a conspicuous research focus, drawing many published papers. Gao et al. proposed an MMA made of ITO and multilayered base, including air and water.^[^ ^56^ ^]^ Through simulation, the authors found that this MMA could absorb 14.4−39.4 GHz range of the EM wave, which had an ultrawide absorption bandwidth compared with ordinary coating materials. Moreover, the ITO–air–water–ITO backplane was also optically transparent and infrared stealth, which was treble‐wave stealth. Its optical transparency was 80% between 400 and 800 nm. It could be concluded that the absorption ability was controllable by ITO pattern design. Another similar work was finished by Yang et al. through three‐layered ITO MMA.^[^ ^57^ ^]^ Three ITO layers were designed into different patterns in a sequential order. The first layer was the infrared (IR) shielding layer consisting of periodic ITO square arrays on glasses. The second layer was the microwave absorbing layer, and the third one was the reflection mirror layer. By treble‐layered ITO, it became possible to design bistealth material with high visible light transparency. The IR emissivity of ITO MMA was 0.15, and it was stable even after being heated to 200 °C.

After being applied in a light field, EMAS materials should be optically transparent, which can be applied in EM windows in aircraft. The low filling ratios of metal nanomaterial film and transparent MMA pattern design are two impactful methods to realize light field responsiveness. At the same time, MMA is a novel way to synthesize multistealth absorbers. Meanwhile, it has still remained a challenge that color‐changing EMAS materials are not considered by researchers yet, which may be a new hotspot. In addition, most of reports are mainly focused on the transparent shielding materials. Thus, the needs of transparent absorbing materials in commercial usage will stimulate the research direction into related systems. Last but not least, metal NWs or NTs are expensive, and how to prepare low‐cost metal‐based EMAS materials with light field responsiveness is still a question.

## 5 Space–Time Field

The time field is a concept in geology originally, describing the transmission of earthquake waves in medium space. Herein, the space–time field is quoted to define EMAS materials with the ability of shape memory or self‐healing, which are variable in space and time. To cut processing and repairing costs effectively, shape memory and self‐healing are two commonly used technologies on aircraft. EMAS materials are usually used as aircrafts surface coating, leading to abrasion by an external force. The satellite antenna is mainly deployed under heat‐inspired shape memory property. This review will focus on possible material systems and fabrication processes about shape memory and self‐healing.

### 5.1 Shape Memory

The shape memory effect is a phenomenon that the material is wholly restored to the original shape before the deformation when heating to a specific temperature; in other words, it can memorize the shape of the mother phase. EMAS materials with shape memory function include films, foams, and papers. The main challenge is how to realize shape memory through molecule design or component control. In recent studies, researchers have devoted themselves to different strategies to realize shape memory and EMAS multifunctional property.

**Figure 6:**
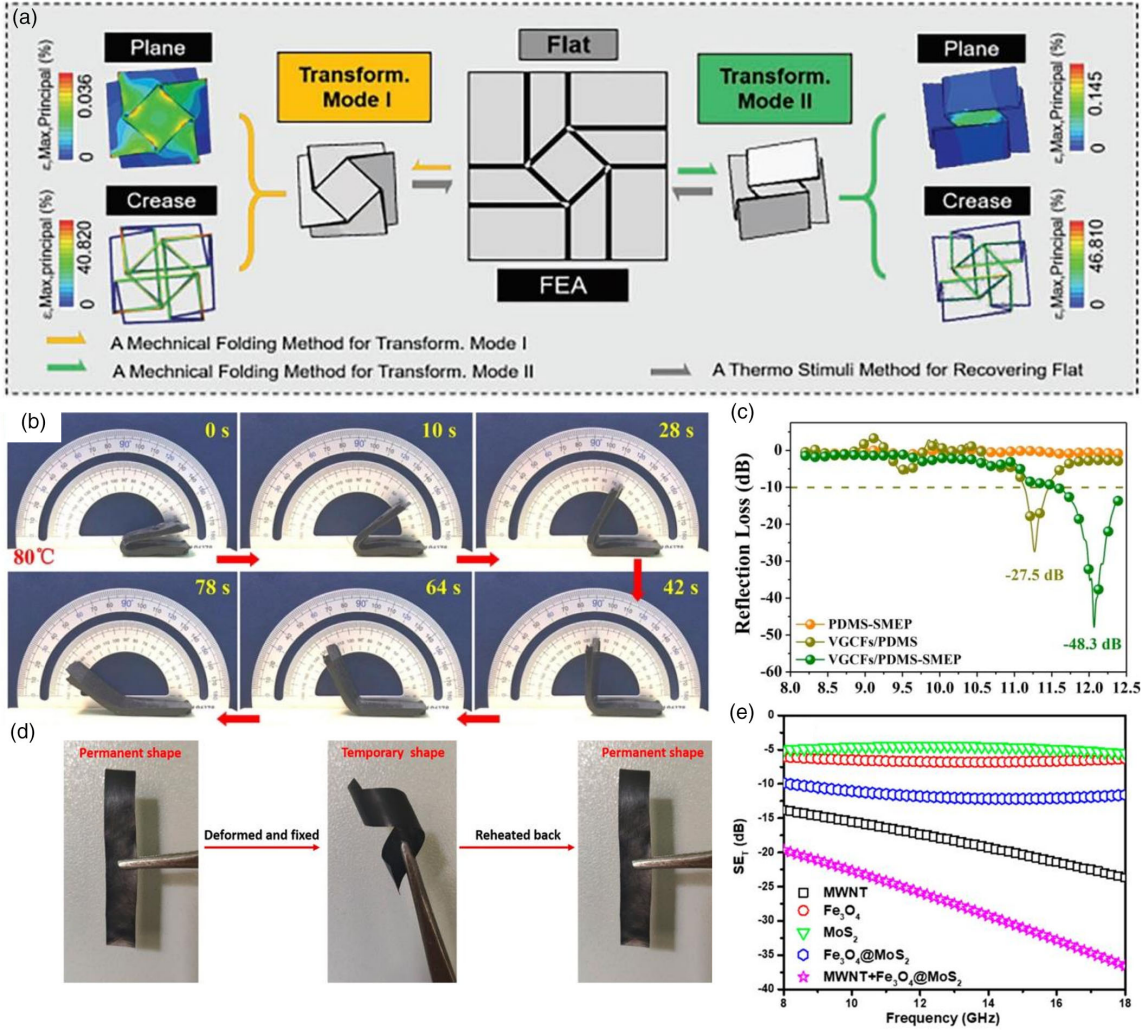
a) The configurations of transformation modes I and II and the strain diagrams. Reproduced with permission.^[^ ^58^ ^]^ Copyright 2020, Wiley‐VCH. b) Shape memory behavior of composites. c) Calculated RL values. b–c) Reproduced with permission.^[^ ^59^ ^]^ Copyright 2020, Elsevier. d) Shape memory property of PU dopamine. e) Total SE. d–e) Reproduced with permission.^[^ ^60^ ^]^ Copyright 2019, American Chemical Society.

As shown in **Figure** **6**a, origami‐inspired structure was designed.^[^ ^58^ ^]^ Origami, like kirigami, is a traditional art in Japan, referring to building a paper model by folding. The square‐twist origami structure was fabricated through 3D printing reported by Wang et al. Two kinds of elastomers were taken into consideration; TangoBlackPlus, soft elastomer, was made into folding lines, whereas VeroWhitePlus, rigid elastomer, was used in a fixed area. The folded origami structure could transform into a flat status in 1 s in two modes by temperature controlling. Its *S* parameters were analyzed using high‐frequency structure simulator (HFSS) software, indicating possible application as an EM wave antenna. Li et al. fabricated vapor‐grown carbon fibers/polydimethylsiloxane–epoxy resin shape memory epoxy sheet (VGCFs/PDMS–SMEP). The prepared composites exhibited a minimum RL value of −55.7 dB at 2.0 mm with an effective bandwidth of 9.8 GHz. Multireflection and dipole relaxation, leading to substantial dielectric loss, were the main reasons for outstanding EM absorbing property. After being folded by force, it would recover by itself within 2 min, as shown in Figure 6b,c.^[^ ^59^ ^]^ VGCFs/PDMS–SMEP could be easily set into different shapes for various application scenes due to its flexibility.

In Figure 6d,e, biomimetic technology was reported by Menon et al.^[^ ^60^ ^]^ A mussel‐inspired polyurethane (PU) dopamine/MWCNT/Fe_3_O_4_@MoS_2_ was proposed as a shape memory EM absorber. The modified PU material was capable of shape recovering after heating to 50 °C. This nanocomposite showed a great EM absorbing property of −36.6 dB, owing to multireflection inside the composite and eddy current losses. The RL values could be manipulated by altering the ratio of every component. This work might be suggestive of electronics flexibility. Wei et al. combined 3D printing and nanofiber‐based materials to realize shape memory property.^[^ ^61^ ^]^ They used silver‐based carbon nanofiber (Ag@CNFs) as 3D printing ink; meanwhile, thermoplastic poly (lactic acid) (PLA) and dichloromethane were considered to be basement and solvent. The model shape could be artificially controlled through software modeling. After being built in a four‐layered structure, its EMI SE was measured as 47 dB in 8.2–12.4 dB. In addition, Wei et al. designed a gripper to realize grabbing function through heating by shape memory property. Yan et al. designed gradient vapor‐grown carbon fiber‐based PU foams as shape memory material.^[^ ^62^ ^]^ At the thickness of 0.11 mm, EMI SE could reach 30 dB, isolating 99% of EM wave. By compression‐modified foams, the electrical conductivity could be manipulated, thus leading to an increased SE value. Due to its double‐layered structure, folding on the positive side has different physical properties from folding on the negative side. Positive bending showed better recovery ability, while negative bending showed better SE values. Kong et al. designed high‐temperature EM shielding material with shape memory using polyimide, 5% carbon fiber, and 4% carbon black.^[^ ^63^ ^]^ By controlling the ratio of carbon fiber and carbon black, tunable EMI SE was obtained. The polyimide film with a thickness of 0.35 mm exhibited 23.9 dB in 8.2–12.4 GHz, whereas its glass transition temperature was 308 °C. The as‐prepared film could lift 2.37 kg weight, and even its mass was only 0.0545 g. The EM shielding value was maintained at 20 dB after 30 reshaping cycles. Similarly, Lu et al. also used carbon fiber as an ingredient to synthesize shape memory composites, combined with poly (ethylene‐*co*‐vinyl acetate) (EVA) for EM shielding.^[^ ^64^ ^]^ Multilayered composites consisting of EVA film and carbon fiber felt were obtained by hot pressing under 150 °C, at 5 MPa. The carbon fiber/EVA composites achieved 45.6 dB EMI SE in X‐band with a conductivity of 16.69 S cm^−1^. While heating after being folded, carbon fiber/EVA composites could recover to their original shape in 30 s, which could be applied in intelligent devices. Apart from carbon materials, MXene is also suitable for shape memory. It was reported that the castor oil‐based WPU/MXene film was applied to grow an ecofriendly shape memory system.^[^ ^65^ ^]^ After 45 °C treatment, the bending and the stretching film would recover to its original shape in 5 min. The authors declared that disulfide band exchange was the possible mechanism of shape memory property. Notably, such a film showed excellent EMI SE of 51 dB, due to MXene component, even after an aging test in 80 °C for 5 days or being bent for 200 times.

Although material component regulation is quite an effective method for establishing shape memory function, structural investigating is necessary. According to Menon's work, a porous architecture was prepared to accomplish shape memory EM shielding.^[^ ^66^ ^]^ It was fabricated using phase inversion and vacuum filtration to coat nanoparticles. The pore size was controlled between 1 and 3 μm. A high EMI SE of 32 dB was realized at the thickness of 400 μm. Different material systems were also studied, including rGO@Fe_3_O_4_, CNT multilayered stack. Furthermore, porous materials with various components exhibited different shape recovery ratios, which reached the highest point of 95% of thermoplastic polyurethanes/rGO@Fe_3_O_4_. Besides film and stack, the foam was widely studied as shape memory materials’ structure. Jia et al. constructed CF composites with trans‐l, 4‐polyisoprene (TPI)‐MXene layer as the coating.^[^ ^67^ ^]^ The wooden foam was pretreated with natural wood and transferred into CF by carbonization. When the filling ratio of MXene reached 20 wt%, EMI SE of composites covered the 8.2–12.4 GHz range at 44.7 dB. The shape memory property of TPI‐MXene/CF was optimized by the Joule heating effect. After being heated to 100 °C, TPI‐MXene/CF recovered to a standard shape in 90 s; on the other hand, with a 3 V external electrical field, the changing time shrank to 15 s. This paper provided a new method to drive shape recovery. Analogously, Zhu et al. adopted foam as the macrocarrier of space–time field‐responsive function.^[^ ^68^ ^]^ The conductive foam was first dipped into a mixing solution of single‐walled CNTs and chitosan salt and then dried at 180 °C for reduction. Shape memory foam was prepared by dip coating using conductive foam. Its EMI SE could reach 56 dB in 8.2–12.4 GHz and be affected by macroscopic formation. When heated in the oven at 75 °C for 5 min, the prepared foam was able to return to its original shape. Through shape memory property, EMAS materials can be folded into small pieces, considerably cutting transportation costs. To further explore the possibility of developing space–time field‐responsive EMAS materials, self‐healing property is reviewed.

### 5.2 Self‐Healing

Self‐healing material can repair itself when surface damage occurs. It effectively cuts the payment of maintaining and repairing, thus widely used in the mechanical engineering domain. Polymers and hydrogels are the most applied self‐healing materials in daily life caused by thermally reversible reactions and ionomeric coupling. In addition, ceramics and metals are also potential candidates. Self‐healable EMAS material is a burgeoning domain, combining chemistry, material science, and mechanics theory. This part will focus on the material system and fabrication procedure of self‐healable EMAS material construction.

**Figure 7:**
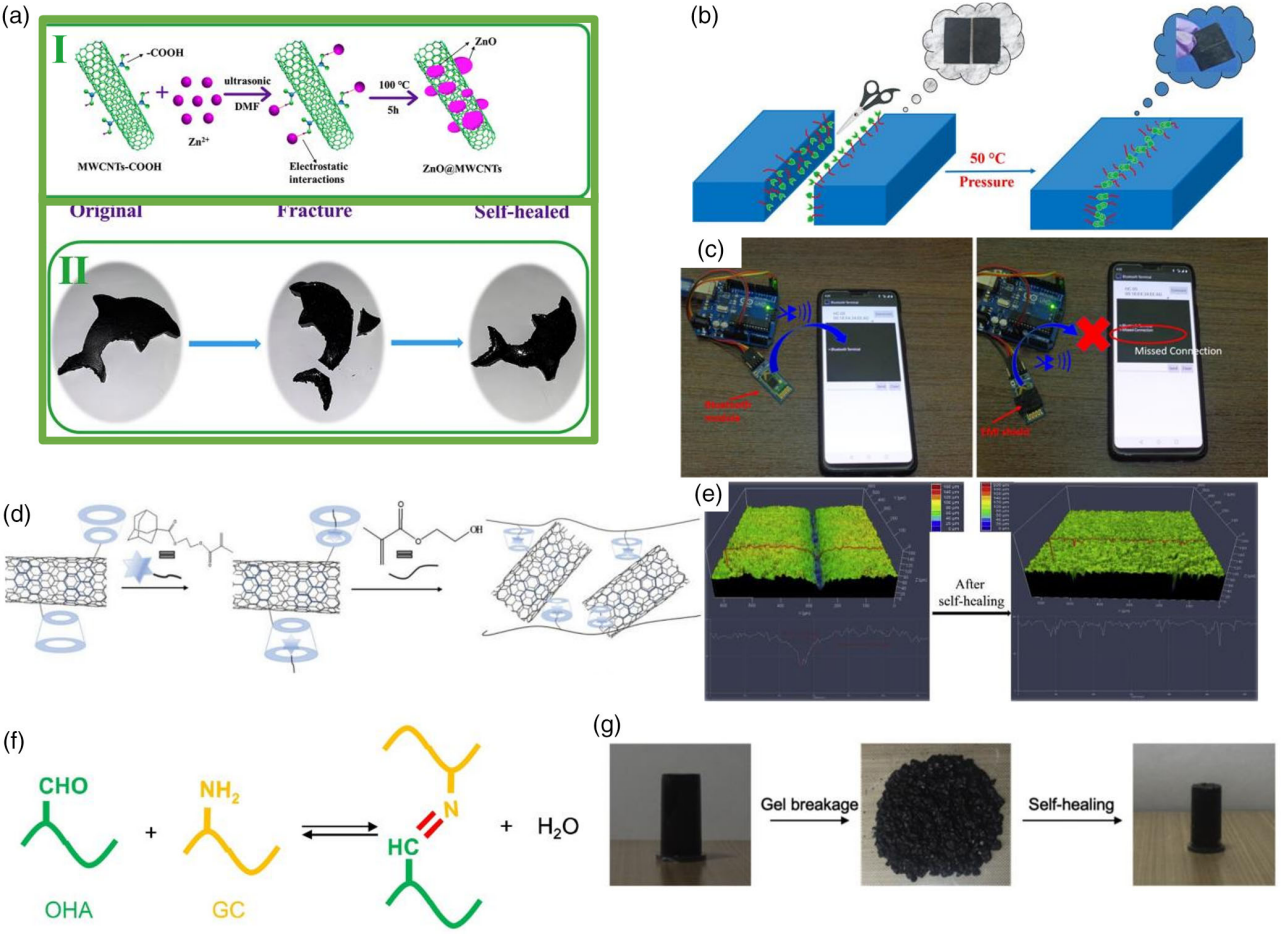
a) Preparation of ZnO@MWCNT nanoparticle and dolphin‐like sample. Reproduced with permission.^[^ ^69^ ^]^ Copyright 2020, Elsevier. b) Schematic representation of self‐healing process. c) Photographic image showing the effectiveness of the developed material. b–c) Reproduced with permission.^[^ ^70^ ^]^ Copyright 2019, Elsevier. d) Schematic illustration of the preparation process. e) LSCM characterization of fabrics before and after scratching. d–e) Reproduced with permission.^[^ ^71^ ^]^ Copyright 2020, Elsevier. f) Schiff base formation between OHA and GC. g) Self‐healing property after gel breakage and self‐healing for 10 min. f–g) Reproduced with permission.^[^ ^72^ ^]^ Copyright 2020, Elsevier.

As shown in **Figure** **7**a, ZnO@MWCNTs/PDMS nanocomposites were proposed as self‐healable EM wave absorbers.^[^ ^69^ ^]^ Herein, PDMS was fabricated by Diels–Alder's (DA) reaction through polymerization of conjugated diolefin and dienophile. Its self‐healing property was verified by cutting it into pieces, and after being maintained together for 10 min at 120 °C, it could connect itself with more than 90% original strength. The 3 mm‐thick ZnO@MWCNTs/PDMS nanocomposites exhibited −20.1 dB absorption in X‐band. Even after being cut off three times, its EM absorbing ability remained nearly unchanged. As shown in Figure 7b,c, Menon et al. developed a thermoplastic polyurethane (TPU)‐based shielding material combined with MWCNTs and MoS_2_/Fe_3_O_4_ nanoparticles.^[^ ^70^ ^]^ It was able to self‐heal at a low temperature of 50 °C. The absorber was constructed into an ultrathin film with the thickness of 200 μm, with an excellent shielding parameter of 3333 dB cm^2^ g^−1^, and EMI SSE/*t* was controllable by adjusting the filling ratio of nanoparticles. When it was covered onto a Bluetooth module, the signal of the Bluetooth device was blocked from a smartphone.

It is shown in Figure 7d,e that a multilayered fabric was fabricated by CNTs–poly (2‐hydroxyethyl methacrylate) (CNT–PHEMA) through electrospinning.^[^ ^71^ ^]^ The CNT network and porous structure were beneficial to EM wave absorption, and the multilayered fabric was applied; thus the prepared fabric could absorb most incident wave even with a very low ratio of CNTs at 0.17 wt%. A laser scanning confocal microscope (LSCM) was used to observe the self‐healing procedure. The crack healed in 24 h under 100% humidity; in the meantime, EM shielding property changed much basically and was restored to 90.86%. Ko et al. considered 3D printing and reported a self‐healable gel, as shown in Figure 7f,g.^[^ ^72^ ^]^ Glycol chitosan (GC), oxidized hyaluronate (OHA), and superparamagnetic iron oxide nanoparticles (SPIONs) were demonstrated to fabricate gel using 3D printing. As shown in Figure 7f, the imine bond was verified in connection with OHA and GC, which was reversible and led to self‐healing. Owing to the introduction of magnetic nanoparticles, prepared OHA/GC/SPION ferrogel would respond to the magnetic field, which might be a potential application in 4D printing.

During the development history of self‐healing materials, various material systems were proposed. Sim et al. fabricated GO/Ag NW composites as healable functional EMI shielding samples.^[^ ^73^ ^]^ The SE of the as‐prepared film was 92 dB, with an ultrathin thickness of 18 μm, which could be regulated by the mass fraction of Ag NWs. This film was synthesized by mixing GO dispersion and Ag NW solution and drying for 24 h naturally. To evaluate self‐healing performance, the GO/Ag NWs were broken and soaked by moisture for 1 h. The EMI SE of GO/Ag NWs was nearly unchanged before and after damage. Wang et al. developed *p*‐(HEMA‐*co*‐BA)‐Fe_3_O_4_ coating through hydroxyethyl methacrylate (HEMA) and butyl acrylate (BA) for EM wave absorption.^[^ ^74^ ^]^ The minimum RL value of this coating was −27.2 dB with broad absorption bandwidth. As‐prepared coating exhibited practical self‐healing ability, and after being cut into two pieces separately, it could recover to its original shape with the help of water. The healed sample only possessed 40% mechanical character of original coating; the author declared that coating material was applied onto the basement surface without being affected by tensile force, which nearly had no side effect. Gels and foams are mostly used macrostructures in the self‐healing domain. Zhu et al. designed a hydrogel consisting of MXene, poly (acrylic acid) (PAA), and amorphous calcium carbonate (ACC).^[^ ^75^ ^]^ Noticeably, this hydrogel exhibited ultrawide bandwidth absorption of 0.2–2.0 THz with EMI SE of 45.3 dB; even the thickness was as low as 0.13 mm. The self‐healing property of prepared hydrogel was highly sensitive due to quick healing time in 1 s. Such gel was easily deformed to build various shapes for application. Accordingly, this work was irradiative to the construction of terahertz functional material. Zhan et al. reported CNTs/acrylic copolymer (AC) foams as self‐healable EM wave shielding material.^[^ ^76^ ^]^ CNTs/AC foam was synthesized by isobutane as a foaming agent and a hot‐pressing process. This unique foam exhibited 1243.3 dB cm^2^ g^−1^ SSE/*t* with only 0.62 vol% CNTs in 8.2–12.4 GHz. After being broken and reshaped, this foam maintained the 79% EM shielding ability of the original one. By being heated to 100 °C, the crack of broken foam would heal in 20 min. In addition, this foam could load a weight of up to 200 g due to appropriate mechanical property.

Self‐healing material is also required in the building industry. Lou et al. studied steel slag asphalt with microwave function and recovery capability.^[^ ^77^ ^]^ Aggregate, ferrite, and steel slag were mixed to improve microwave heating uniformity. The minimum RL value of composite filler was −30.28 dB between 12.91 and 13.92 GHz. The self‐healing ratio was studied by semicircular bending; results showed that the crack slowly healed itself by 0.5 mm min^−1^ and was controllable by the ratio of mixtures. This technology was beneficial to the construction industry with multifunction. Ma et al. prepared a 3D hollow structure using benzotriazole (BTA) and NiCo_2_O_4_ through hydrothermal reaction for EM wave absorption.^[^ ^78^ ^]^ A 2 mm‐thick coating composed of BTA@NiCo_2_O_4_ exhibited −35.39 dB with an effective absorbing bandwidth of 4.64 GHz because of interfacial polarization and dipole polarization. Its self‐healing performance was realized due to the BTA release. After a scratch was made on the coating surface, the maximum depth of it decreased from 15.756 μm gradually to 2.29 μm.

Extensive researches have confirmed that the space–time field applied to EMAS materials can multiply application proportion. Carbon materials and resin polymer are mainly used materials systems, while foam, film, and gel are frequently discussed. The shape memory material is widely used to construct skin‐like films or compressible and elastic 3D blocks, while self‐healing material is used in gels repairment or coating scratch recovery. 3D printing technology is also investigated for its potential value in macro‐space–time field‐responsive material synthesis. Yet, there are still many problems that remained. For instance, many shape memory materials have the shortage of too few cycle times, irreversible change, or long changing time; self‐healing materials, in contrast, face the defects of long healing time, high reaction temperature, or low percentage of recovered function. Thus, it is urgent that researchers should develop a quick, responsive and multicycled space–time field‐responsive material.

## 6 Electrical Field

EMAS materials are either electrically conductive or dielectric active, and both will be affected by an external electrical field. In this part, typical dielectric materials and relative dielectric constant are reviewed at the status while being affected by the electrical field. Directional movement of absorption peaks can be realized if EM parameters can be controlled accurately, leading to controllable EM absorption band ranges. Low‐frequency absorption may be achieved easily through the external electrical field method. The effect and mechanism of external electrical fields manipulating EM parameters are reviewed in detail.

**Figure 8:**
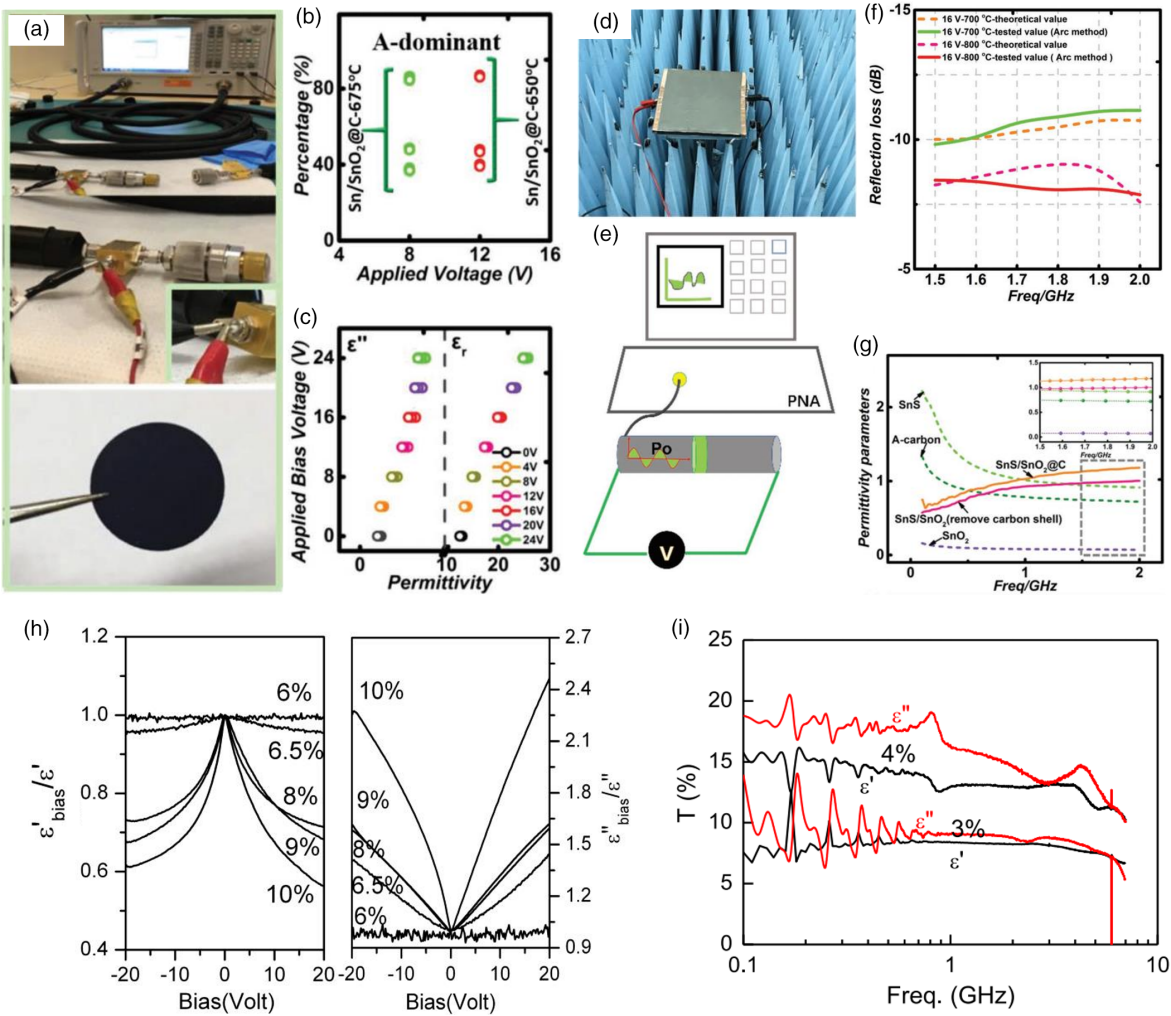
a) The schematic illustration of the single coaxial‐line method. b) The comparison of *S*, *A*, and *R* values of the Sn/SnO_2_‐650 °C and Sn/SnO_2_@C‐675 °C at the applied voltages of 12 and 8 V. c) The *ε*"/*ε*_r_‐voltage trends. a–c) Reproduced with permission.^[^ ^79^ ^]^ Copyright 2019, Wiley‐VCH. d) The setup of the NRL arc. e) The schematic illustration of the single coaxial‐line method. f) The RL of this device tested by the coaxial‐line and NRL‐arc methods. g) Dielectric loss value of SnS/SnO_2_@C, SnS, SnO_2_, SnS/SnO_2_, and amorphous carbon. d–g) Reproduced with permission.^[^ ^80^ ^]^ Copyright 2018, Wiley‐VCH. h) Tunability of permittivity at 1 MHz. Reproduced with permission.^[^ ^81^ ^]^ Copyright 2008, AIP Publishing. i) Tunability of the MWCNT composite membrane with different concentrations at a bias field of 10 V mm^−1^. Reproduced with permission.^[^ ^82^ ^]^ Copyright 2012, Springer Nature.

As shown in **Figure** **8**a–c, our group designed an EM wave shield using graphene film.^[^ ^79^ ^]^ Graphene film was used as a central layer and covered by Sn/SnO_2_@C from both sides (SD) with different Sn contents. After the material structure was constructed, it was combined with an external electrical field. By turning external voltages, the specific frequency was able to be controlled to 8–8.4, 8–9.3, or 8–10.3 GHz frequency range. Through combining material science and bias voltage, tunable transmission ability was realized. Furthermore, in Figure 8d–g, we proposed a voltage‐boosting method to construct an EM absorption device.^[^ ^80^ ^]^ Two strategies, including materials synthesis and external electrical field, were applied to build an EM wave absorption device aiming at low‐EM frequency. SnS/SnO_2_@C was used as the middle layer, covered by two electrolytes. After external voltage was applied, the prepared absorber could absorb more than 85% of the EM wave in 1.5–2.0 GHz. More than that, owing to the thermal stability of the aforementioned device, the EM absorption ability could nearly stay unchanged even after being heated to 150 °C. Our work provided a novel strategy for high‐temperature and low‐frequency EMAS devices’ design.

It is shown in Figure 8h that Liu et al. prepared low‐filling‐ratio CNTs at only 6 wt% and studied its dielectric parameters while it was put in external bias voltage.^[^ ^81^ ^]^ As the applied voltage increased, the real part of complex relative dielectric parameter decreased and its imaginary part increased. The tunable ability of CNTs was mainly caused by free electrons in the network formed by CNTs. The change of the dielectric parameter is the basement of the controllable EMAS property. In Figure 8i, Liu demonstrated a new work.^[^ ^82^ ^]^ Silicone composites, combined with MWCNTs, were synthesized by a spinning and coating procedure. After that, composites were shaped into a membrane, and its permittivity was studied in the frequency range of 0.1–7 GHz by single‐port testing. The relative permittivity varied with the change of MWCNTs’ ratio and external voltage. Using bias voltage, the relative permittivity was improved in the gigahertz range. The as‐prepared composites membranes paved the way for synthesizing controllable EMAS properties.

The EMAS ability in UHF, like the terahertz range, also can be affected by an external electrical field. Chen et al. designed a metamaterial using graphene and used metallic grating to build a metasurface.^[^ ^83^ ^]^ The external voltage was small between −2 and 2 V; still, it could manipulate the amplitude of as‐prepared MMA, and the best absorption ratio was 23 dB. Through the novel methods of metamaterial and external electrical fields, terahertz range absorption at 0.2–1.0 THz was realized effectively.

The electrical field‐led field‐responsive material is a rising domain without many scientific reports, which is lifeful and worth digging deeper into. By applying an external electrical field, relative permittivity can be changed, resulting in EMAS ability controllable. Terahertz absorber can be constructed through bias voltage. Metallic oxide and carbon material, which are reactive to the electrical field, constitute the current electrical field‐responsive material system. The following researchers should focus on the mechanism and new macrostructure design to expand possible contents.

## 7 Wind Field

The wind field is an environmental impact assessment professional terminology used in quantitative analysis of the wind speed. In the procedure of laboratory materials science industrialization, several durability tests must be conducted to verify its possibility, including the erosion–corrosion resistance test, which is run by high‐temperature wind flow containing abrasing particles. In that case, EMAS materials have to stabilize under the vectorial wind field. The negative effects caused by wind field can be divided into two parts, flame and wear and tear, requesting that EMAS materials be flame retardant and abrasion resistant. Herein, the common wind field‐responsive materials are reviewed and their structures are analyzed.

### 7.1 Flame Retardancy

Flame retardancy is a vital property for wind field‐responsive EMAS material. The aforementioned heat insulation property in Section 3.2 is a similar concept compared with flame retardancy. Nevertheless, it should be emphasized that they are two different performances of functional materials, even though they are frequently confused by researchers merely because a part of materials is compatible in flame retardancy with heat insulation. Flame retardancy means that a subject cannot be lit by fire, while heat insulation refers to low thermal conductivity. Take a simple example of paper and iron bars. Paper is a heat isolation material, ordinarily used in catering industry takeaways in case of scald, but paper can be lit by fire easily. Meanwhile, the iron bar has a high thermal conductivity but is unable to burn.

**Figure 9:**
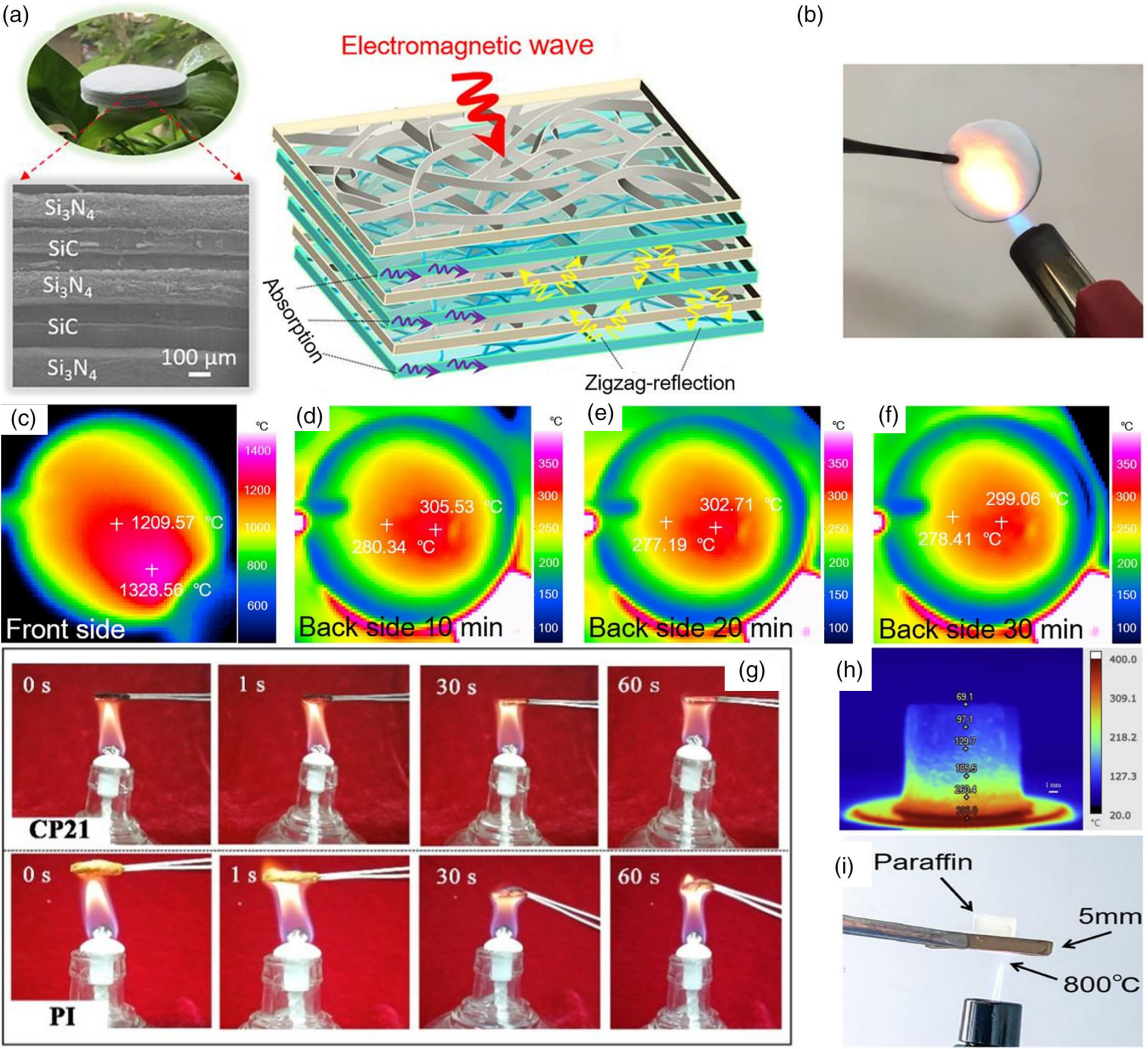
a) Digital photograph of aerogel floating on a leaf and its structure. b) Optical photograph of the aerogel during ablation. c) Front‐ and d–f) back‐side thermal infrared images of the aerogel at 10, 20, and 30 min, respectively. a–f) Reproduced with permission.^[^ ^84^ ^]^ Copyright 2021, American Chemical Society. g) Digital photographs of two foams on the flame of an alcohol burner with time ranging from 0 to 60 s. Reproduced with permission.^[^ ^85^ ^]^ Copyright 2020, American Chemical Society. h) Infrared image of graphene aerogel on a 400 °C heating stage for 1 h. Reproduced with permission.^[^ ^86^ ^]^ Copyright 2018, Elsevier. i) Photograph of SiC foam burning at 800 °C. Reproduced with permission.^[^ ^87^ ^]^ Copyright 2021, American Chemical Society.

As shown in **Figure** **9**a–f, Cai et al. designed a multilayered EM wave absorber consisting of Si_3_N_4_ and SiC alternately.^[^ ^84^ ^]^ Due to the millimeter‐scale space in Si_3_N_4_/SiC aerogel, multireflection emerged, causing a broad absorption band for 8.4 GHz. The minimum RL value of alternate Si_3_N_4_/SiC aerogel was −45 dB. Lit by a flame gun, the as‐mentioned aerogel was heated to more than 1300 °C in the front. After being baked for 30 min, this material was still stabilized, showing an excellent flame resistance.

In Figure 9g, the flame retardancy of CNTs/polyimide (PI) foam was tested on the flame of an alcohol burner.^[^ ^85^ ^]^ After burning for 60 s, the main structure of CNT/PI foam remained complete, which was remarkable as carbon material compared with others like carbon cloth. Internal carbon skeleton provided a connected conductive network for electrons, bringing 41.1 dB EMI SE. Furthermore, the absorption coefficient of CNT/PI foam was 82.3%, which was quite rare in a high‐conductivity material system and caused by plenty of interfacial polarization. There were other carbon materials used as fireproof agents. Wang et al. developed graphene aerogel as an EM wave absorber, as shown in Figure 9h.^[^ ^86^ ^]^ The graphene aerogel was fabricated by in situ assembly method using GO as raw material and was shaped by freeze‐drying. Final aerogel was reduced in a pipe furnace for 2 h. This graphene gel exhibited a RL value of −61.09 dB at 4.81 mm with an effective absorption bandwidth of 6.30 GHz. To verify its fireproof property, graphene gel was put on the 400 °C fire for 1 h. Its EM absorption was changeless before and after burning. Foams and gels are commonly used as inflaming retarding materials. As shown in Figure 9i, Su et al. fabricated a SiC_nw_@SiC foam by chemical vapor deposition (CVD).^[^ ^87^ ^]^ Melamine foam was reduced into C foam, and through the CVD method, SiC was grown onto the surface of C foam. SiC foam was further obtained by oxidation to remove the C skeleton. SiC_nw_@SiC was finally obtained by CVD again. The best RL value of the as‐mentioned foam was −52.49 dB with a 5.6 GHz wide absorption band. This foam was unchanged, even after being heated by an 800 °C flame.

CVD is widely applied in the semiconductor industry to manufacture coatings or nanoparticles. Li et al. also used CVD to grow carbon‐coated carbonized loofah sponge (CCLS)/MXene aerogel.^[^ ^88^ ^]^ Dried chalina was cut into sponge pieces, and the latter was further prepared for CCLS through phenolic resin and carbonization. CCLS/MXene aerogel was built by CCLS and MXene solution using directional freeze‐drying. CCLS/MXene aerogel could be maintained in the fire for at least 120 s. The directional MXene arrangement and microporous structure were beneficial to EM wave shielding, which was 70.0 dB. Li's work united biomass, MXene material, CVD, and directional aerogel, which was inspiring for correlative research.

MXene‐based materials also have been widely used in fire‐resistant functional material synthesis. Zhang et al. reported an aerogel consisting of CNF, ammonium polyphosphate (APP), and MXene.^[^ ^89^ ^]^ This kind of CNF/APP/MXene aerogel was fabricated by the freeze‐drying method with different APP component ratios from 0% to 40% as the variable. When the aerogel was 8 mm thick and APP content was 40%, the best EMI SE value reached 55 dB. To verify its flame retardancy, as‐prepared aerogel was placed on the burner flame for 60 s. The volume of burnt aerogels stayed unchanged. It was reported that montmorillonite (MMT) and MXene were combined to obtain an EM shielding film.^[^ ^90^ ^]^ The film was shaped by mixing MMT and MXene solution followed by vacuum filtration. After being burnt in the flame for 120 s, the MMT/MXene film phase remained the same as the original one, concluded from X‐ray photoelectron spectroscopy (XPS) analysis. The film composites exhibited an EMI SE of 67 dB and an excellent Joule heating effect. In addition, the SSE/*t* of the above film was calculated as 10 000 dB cm^2^ g^−1^. MMT contains a nonflammable particle TiO_2_, and similar composites with oxide constituents have been reported. Yuan et al. designed a hybrid fiber with Fe_3_O_4_ and SiO_2_.^[^ ^91^ ^]^ The composite fiber was fabricated by the sol–gel method and electrospinning. SiO_2_ was used to optimize impedance matching degree. The minimum RL value was −20 dB at the thickness of 2.5 mm with an absorption band wider than 6 GHz. Its flame‐resistant property was verified by flame treatment. Furthermore, as‐prepared Fe_3_O_4_/SiO_2_ hybrid was confirmed via acid proof by putting in acid for 6 h and ending up with magnetism. Liang et al. reported coating with fire‐resistant property.^[^ ^92^ ^]^ Melamine polyphosphate (MPP), graphite nanoplates (GNPs), and acrylic were used to fabricate fire‐proof coating by stirring and stoving. The filling ratios of MPP and GNPs were two variables applied to manipulate EMI SE of the above coating. The best EMI SE this coating could have was 15 dB. Due to the fire‐proof property of GNPs/MPP/acrylic coating, it could be used to protect ancient wooden buildings, in case the tragedy of Notre Dame de Paris occurs once more. The fire lit on the wooden sample with the above coating would die away in 38 s. Qu et al., in contrast, proposed a new strategy using the covalent bonds to connect components.^[^ ^93^ ^]^ Black phosphorene (BP)–MWCNTs were fabricated by BP–NH_2_ and MWCNT–COOH. The possible mechanism of BP–MWCNTs’ flame retardancy was that the composite film would burn first and protect primary material. Phosphorus element would become HP_*x*_O_*y*_ and reduce flammable materials. Nitrogen element was burnt into N_2_, which was nonflammable. MWCNTs became a new carbon source to be burnt, replacing flammable basement. With the synergistic effect, BP–MWCNTs exhibited excellent flame retardancy. With the help of flame‐resistant composites, EMAS materials can endure high temperatures brought by the wind field. To better accomplish materials responsive to the wind field, abrasion‐resistant property is summarized.

### 7.2 Abrasion Resistance

Abrasion‐resistant materials are pretty significant for the EMAS domain. Ordinarily, EMAS devices are applied in an open place, which will be damaged by corrosion and abrasion. If the outer layer of EM devices is broken, the internal absorber, like MXene, ferrite, or alloy, may be oxidized and EM property will decrease. Many researchers have devoted themselves to developing abrasion‐resistant material to prolong the service life of EMAS coating. Herein, macro‐EMAS materials, like ceramics and concrete, are concluded as follows.

**Figure 10:**
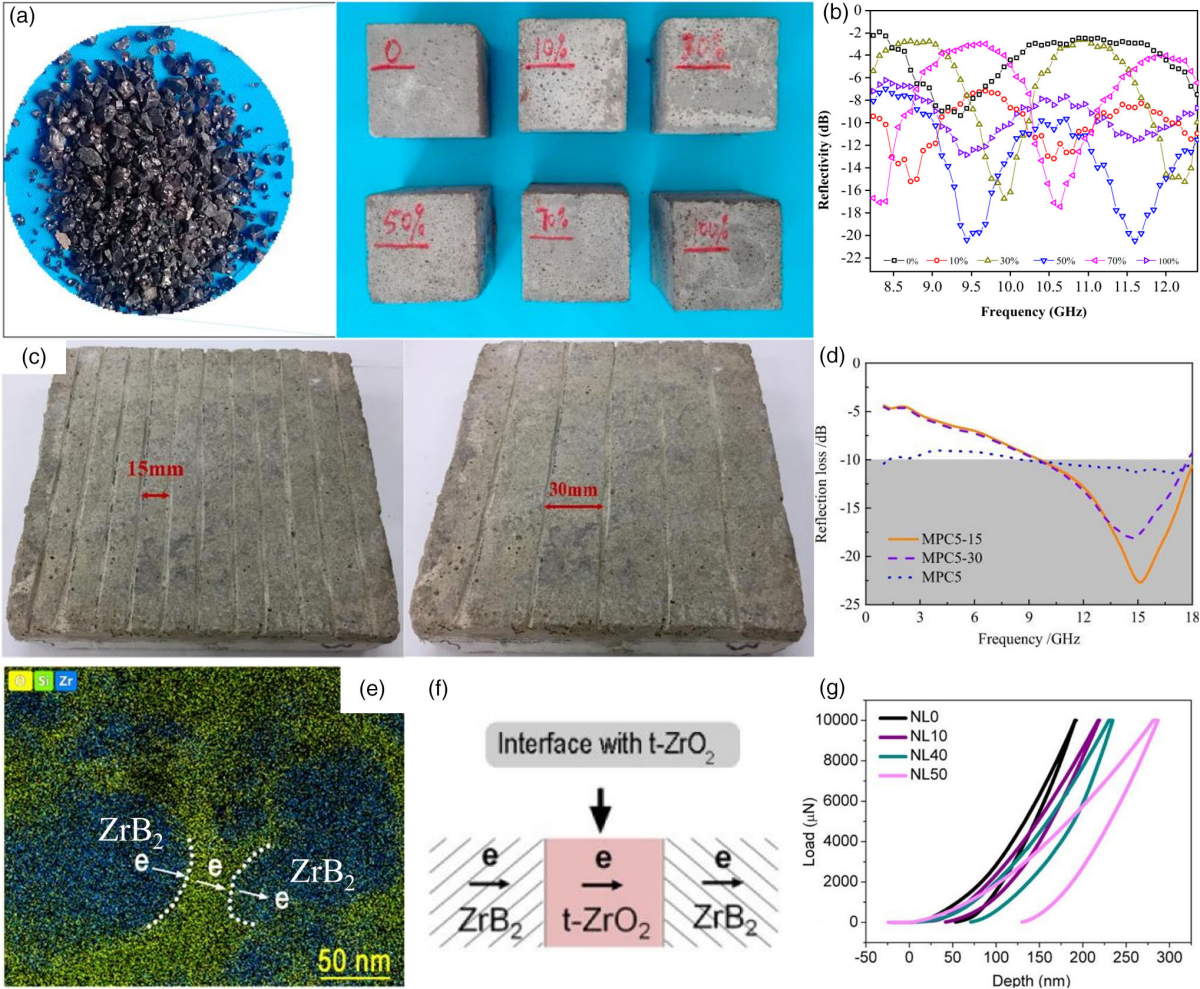
a) Appearance of specimens with different contents. b) Reflectivity of specimens with different contents. a–b) Reproduced with permission.^[^ ^94^ ^]^ Copyright 2021, Elsevier. c) Specimens with notches. d) RL of MPC with different notches. c–d) Reproduced with permission.^[^ ^95^ ^]^ Copyright 2020, Elsevier. e) Energy dispersive spectroscopy mapping of the PDC with ZrB_2_. f) Schematic illustration of interfaces. g) Load–depth curves of the samples during nanoindentation. e–g) Reproduced with permission.^[^ ^96^ ^]^ Copyright 2020, American Chemical Society.

As shown in **Figure** **10**a,b, Guo et al. combined Cu slag and cement and designed macrobulk composites.^[^ ^94^ ^]^ Different volume contents of Cu slag were added to the water/cement mixture and solidified simultaneously. When the filling ratio of Cu increased, more pores would occur in the bulk composites, causing low durability. Meanwhile, the modest increment of Cu contents brought dielectric loss with a more minor reflection wave. Based on this, Guo proposed that 50% Cu slag added in the composites was the most beneficial condition. At 50% Cu ratio, the RL value of bulk composites reached −21 dB. Ma et al. reported a cement composite combined with Fe_3_O_4_ nanoparticles to amplify magnetic loss, shown in Figure 10c,d.^[^ ^95^ ^]^ By the introduction of hollow glass microspheres and notches on the surface of cement with different spacings, the RL value was further decreased. When the distance of two neighboring notches was 15 mm, the minimum RL value was −26.4 GHz. Fe_3_O_4_ nanoparticles, along with hollow glass microspheres, made early strength higher, significantly improving compressive and flexural strengths. The highest flexural strength could reach more than 11 MPa, and compressive strength exceeded 42 MPa.

A ceramic composite was also proposed to shield EM waves in Figure 10e–g.^[^ ^96^ ^]^ Jia et al. synthesized a polymer‐derived SiOC ceramic (PDC‐SiOC) and combined it with ZrB_2_. The composite was prepared by solution mixing and ball milling, followed by pressing and high‐temperature pyrolysis. As shown, electrons transferred between ZrB_2_ and t‐ZrO_2_, leading to interfacial polarization and increasing EM wave loss. EMI SE was measured at 72 dB in 26.5–40 GHz. As‐prepared ceramic provided a new strategy to fabricate abrasion‐resistant EMAS materials. Silicon compound is widely adopted in high‐hardness material, owing to covalent bonds inside the atomic crystal. Li et al. fabricated a SiC foam as an EM wave absorber.^[^ ^97^ ^]^ It was prepared by sintering at air condition with 1200 °C‐close roaster using Si_3_N_4_ and other mineral powders. The as‐prepared bulk foam had a density as low as 0.48 g cm^−3^. The EM wave absorption ability of SiC foam was tested; the minimum RL value was −11.9 dB in a wide frequency range of 8.0–18.0 GHz. In addition, SiC foam also possessed high compressive strength, and its EM wave absorption was stabilized even at 1000 °C. Similarly, porous edge‐rich graphene (ERG)/Si_3_N_4_ ceramic was brought in for EM wave absorbing by Ye et al.^[^ ^98^ ^]^ Si_3_N_4_ was prepared by low‐pressure sintering, and ERG was fabricated by CVD using CH_3_OH in detail. The lowest RL value of ERG/Si_3_N_4_ composites was −23.5 dB at the thickness of 4.75 mm of 9.27 GHz with an absorption band of the 4.2 GHz range. Due to the protection of Si_3_N_4_, graphene was prevented to oxidation, and abrasion‐resistant property was ensured.

In a word, flame retardancy and abrasion‐resistant property are keys to ensure EMAS materials stabilization in the wind field. These two features hugely prolong the life length of EMAS coating while facing a harsh environment in the open air. A wind field‐responsive material is of vital importance to spacecraft when it re‐enters the atmosphere from aerospace. Recent studies mainly focus on silicon compounds and cement, along with fireproof foam or aerogel. Abrasion‐resistant absorbers should be enriched. Absorption values of current composites are not very satisfactory; the following researchers should develop more potent absorbers with a wide absorption bandwidth. Moreover, these abrasion‐resistant absorbers face the shortcoming of high thickness. Few of them even reach the thickness of tens of centimeters. Reduction of applied thickness is also a necessary research domain.

## 8 Density Field

EM absorbing material is evaluated by two main parameters as mentioned earlier. One is *Z*_in_ and another is RL. To ensure that EM waves penetrate the air–material interface as much as possible, *Z*_in_ should be close to 1. In contrast, RL should be as small as possible. However, low reflection is conflicting with solid absorption, which is already determined by relative complex permittivity and permeability in theory. Thus, how to realize a harmonious equilibrium between reflection and absorption becomes an actual problem. One of the effective methods is a multilayered graduated impedance structure. Some of the outer layers play a role in impedance matching to transmit incident waves, whereas inner layers are used as absorption materials. The above solution can be concluded as density field‐responsive material design due to the different density along the vertical direction.

**Figure 11:**
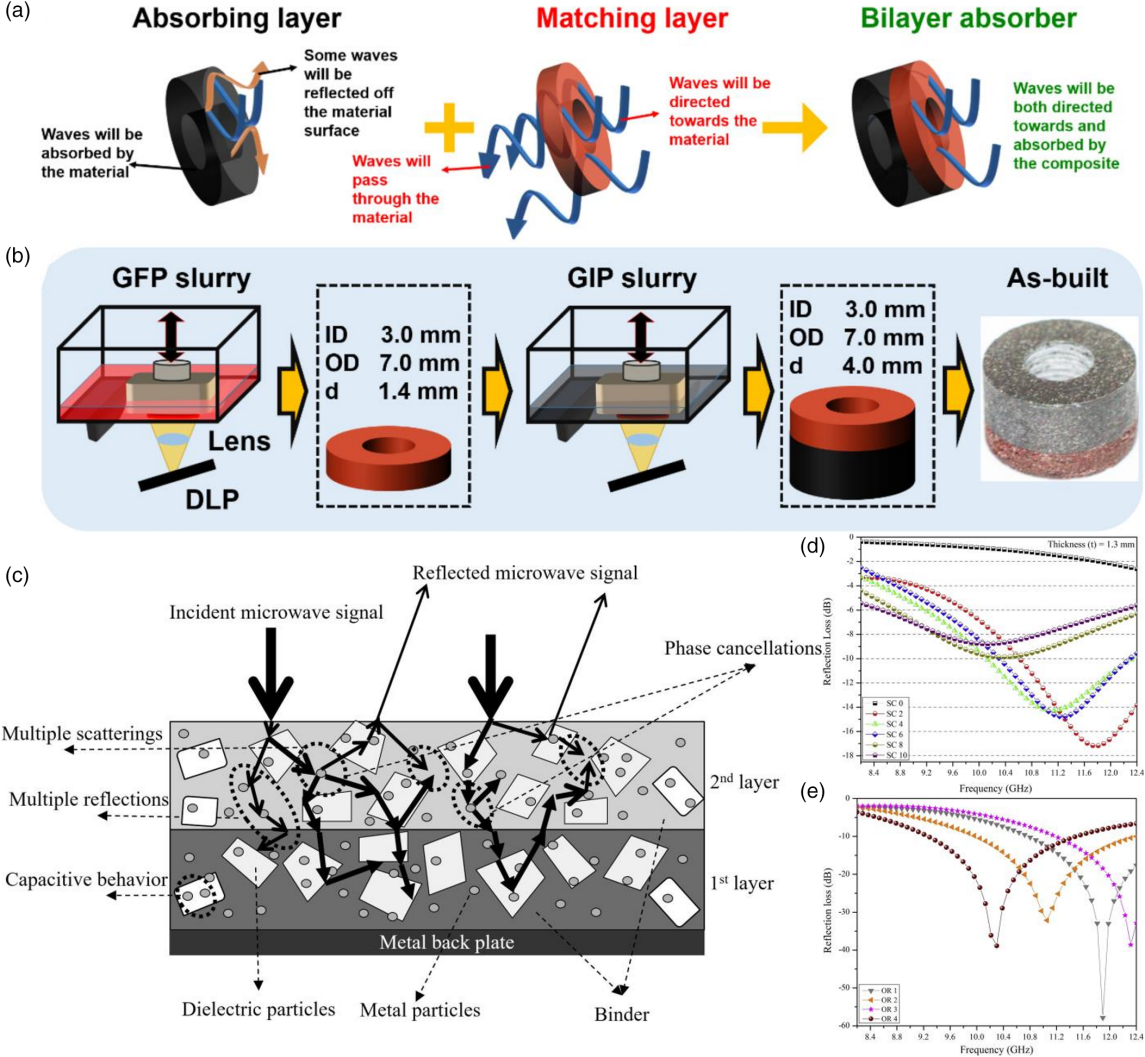
a) Schematic diagram of the design principle of bilayer absorber. b) Schematic diagram of the DLP 3D printing process of the GFP + GIP bilayer absorber. a–b) Reproduced with permission.^[^ ^100^ ^]^ Copyright 2020, Elsevier. c) Schematic representation of microwave interactions. d) RL curves for the Cu‐dispersed SiC composites. e) RL curves for GA‐optimized Cu‐dispersed SiC composites. c–e) Reproduced with permission.^[^ ^101^ ^]^ Copyright 2018, Elsevier.

Usually, a multilayered structure is complex to synthesize. However, with the development of additive manufacturing, or 3D printing, the multilayered graduated impedance structure can be constructed easily. 3D printing is a technique of constructing objects using adhesive materials such as light‐cured resin to build powder metal or plastic into designed macrostructure and printing layer by layer.^[^ ^99^ ^]^ Light‐cured resin is a resin sensitive to UV light and coagulates if radiated by UV light. By mixing and radiating absorbing materials with light‐cured resin, layered plates can be synthesized in minutes or hours. Digital light processing (DLP) 3D printing is a widely used additive manufacturing method. As shown in **Figure** **11**a,b, Zuo et al. designed a double‐layered EM wave absorber consisting of graphene/Li_0.35_Zn_0.3_Fe_2.35_O_4_ (GFP) and graphene/carbonyl iron powder (GIP), and polymethyl methacrylate was used as the basement.^[^ ^100^ ^]^ GFP was the matching layer while GIP was the absorbing layer. Different thickness combinations of GFP/GIP composites were researched, and the best group was selected. Through optimization, bilayers were decided as 1.4 and 2.6 mm. The composites had two absorption peaks of −46.1 dB at 4.7 GHz and −17.9 dB at 15.7 GHz; the total effective absorption bandwidth was 3.5 GHz.

In Figure 11c–e, another bilayer EM absorber was reported by Singh et al.^[^ ^101^ ^]^ SiC was selected as the matrix, and Cu particles were used to optimize its matching and absorbing ability. By ball milling, the two‐layered structure was synthesized. The first layer served as a capacitor and produced interfacial polarization, whereas the second layer introduced multiple scattering and multiple reflections. The bilayer absorber exhibited a RL value of −32.16 dB with a thickness of 1.67 mm and a 2.35 GHz absorption band. Rezazadeh et al. fabricated a double‐layer nanocomposite for EM wave absorption.^[^ ^102^ ^]^ Composed of polypyrrole (PP) and natural rubber (NR) with the ratio of 15:85, the matrix was proposed. The first matching layer consisted of graphite, Fe_3_O_4_, and TiO_2_; meanwhile, the second layer was made of Fe_3_O_4_. The optimum solution was 4 wt% graphite and 15 wt% Fe_3_O_4_, with a −32 dB RL value and more than 10 GHz bandwidth. Zhang et al. fabricated a NiCo_2_O_4_ nanofiber and used it as an ingredient to design a multilayered strain sensor.^[^ ^103^ ^]^ NiCo_2_O_4_ nanofiber was synthesized by electrospinning and calcination. As‐prepared composites possessed a minimum RL value of −52.72 dB. If the multilayered sensor was compressed by force, EM absorbing property would change as well; thus, by monitoring reflection spectra, the structural strain could be perceived accurately. This work provided a novel strategy to design intelligent devices through multilayered EM‐responsive composites.

Density field‐inspired EMAS materials have the ability to combine excellent impedance matching and strong absorption by dividing different functions into various components. Furthermore, the outer layer can also be designed with other multifunctions, like antioxidation, fire resistance, or specific shape with low‐radar‐scattering areas. However, only a few papers reported about it, and the calculation mechanism of RL and *Z*_in_ should be updated as current formulas are deduced by a single‐layered metal backplane model.

## 9 Flow Field

Flow field refers to space where velocity and pressure are variable with coordinates in physics, defined by the Euler method. In this part, it is quoted to describe water flow field in specific. Considering that EMAS materials usually are overlaid on the surface of electronic devices, the interfaces between materials and air are easily covered by dirt, which may lead to diffuse reflection and worse absorbing or shielding. Thus, the self‐cleaning property is valuable to maintain neat materials when EMAS materials are in the water flow field.

**Figure 12:**
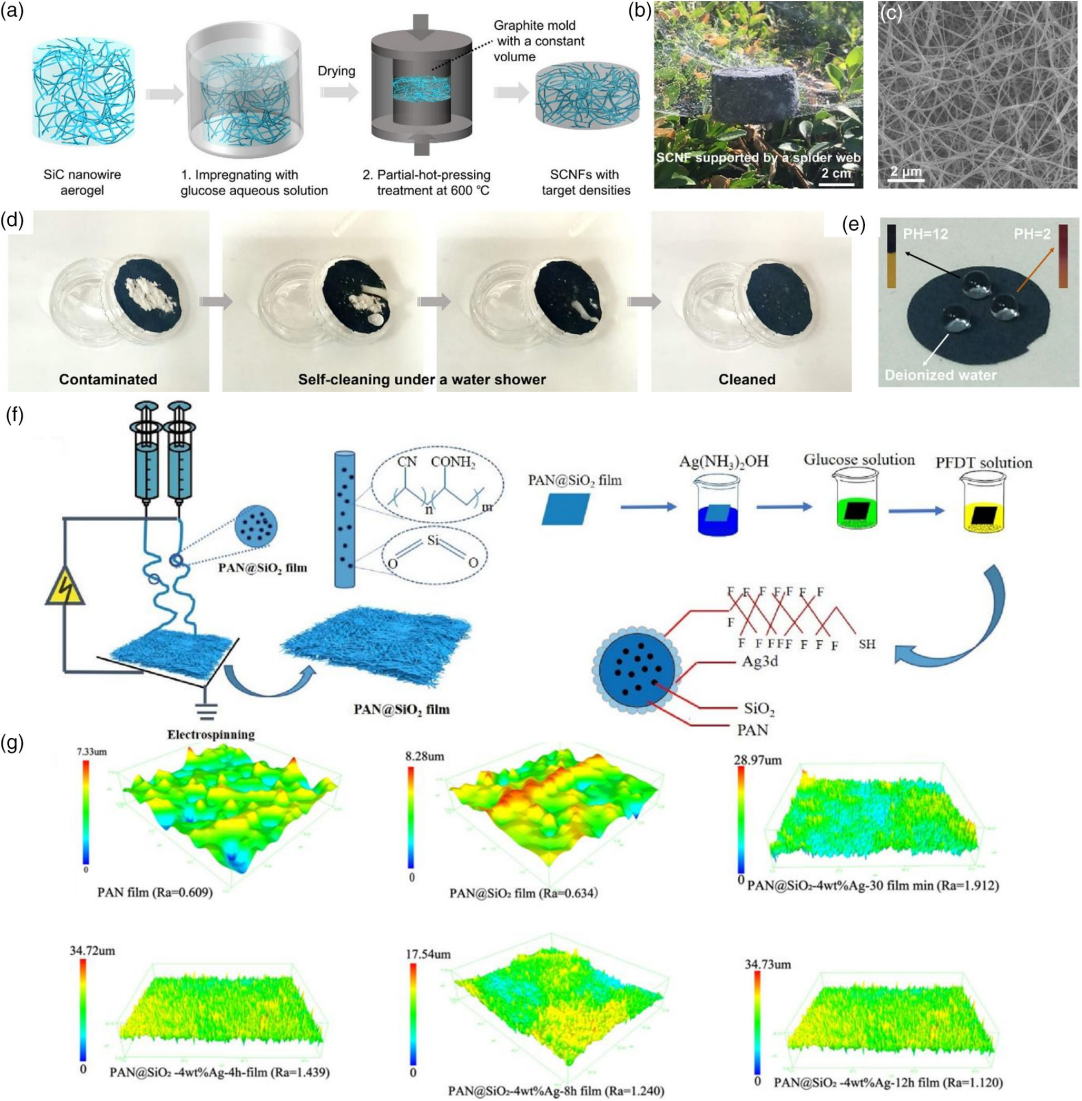
a) Schematic illustration of fabricating the amorphous carbon shell on the SiC nanowires. b) Digital photograph of SCNF036 supported by a spider web. c) SEM image of the SCNF with microscale pores. d) Demonstration of the self‐cleaning performance of the SCNF. e) Optical images of water drops with different pH values on the surface of the SCNF. a–e) Reproduced with permission.^[^ ^104^ ^]^ Copyright 2020, American Chemical Society. f) Schematic illustration for preparation of the composite film. g) Effect of dipping time on the roughness of conductive composite films. f–g) Reproduced with permission.^[^ ^105^ ^]^ Copyright 2020, Elsevier.

As shown in **Figure** **12**a–e, Cai et al. first developed SiC nanowire aerogel by the CVD method and obtained SiC@C nanowire foams (SCNFs) through hot pressing.^[^ ^104^ ^]^ As‐prepared SCNFs exhibited excellent absorption ability with a minimum RL value of −52.5 dB and an ultrawide effective band range of 15.1 GHz between 2.9 and 18 GHz. The EM wave loss was mainly caused by induced current and defect and interfacial polarization at the SiC–C interface. Contamination could be cleaned by dropping water onto the foam, which was self‐cleanable. Apart from foam, the film also shows normal formation for self‐cleaning development. Li et al. reported a polyacrylonitrile (PAN)@SiO_2_−Ag film with ultrahydrophobic character, as shown in Figure 12f,g.^[^ ^105^ ^]^ PAN@SiO_2_ film was first synthesized by electrospinning; then, silver mirror reaction was applied to grow Ag nanoparticles, and PAN@SiO_2_−Ag film was finally obtained by being dipped in 1H, 1H, 2H, 2H‐perfluorodecanethiol (PFDT) solution. The above film showed an excellent shielding property with a 73 478 dB cm^2^ g^−1^ SSE/*t* value, owing to the multireflection introduced by the multilayered structure. To verify its hydrophobicity, the roughness affected by the dipping time of the as‐prepared film was measured. The surface of this film was able to remain smooth even being dipped in water for 30 h. Bioinspired metamaterial is also a potential candidate as water flow field‐responsive material. Huang et al. got inspired by moth eye and constructed a bilayered hexagon array metamaterial.^[^ ^106^ ^]^ The effective absorption band was 8.04–17.88 GHz, covering X and Ku bands. In addition, the infrared emissivity of bioinspired metamaterial was able to reduce. Through microscale size controlling, the hydrophobicity of samples could be accurately adjusted. When the edge of the hexagon was 50 μm long, the contact angle was nearly 135°. Furthermore, this sample realized visible light scattering as well to accomplish camouflage color changing.

In summary, hydrophobicity is a vital character to realize self‐cleaning, and only in this way can EMAS materials stabilize themselves under the flow field. Many researchers have concentrated on hydrophobic foam, aerogel, or film. Yet, the most frequently used EMAS material form is coating. The hydrophobic coating should become the research center. Another question, which is easy to be ignored, is what will happen to EMAS property if a composite cannot clean itself. Many published papers only focused on how to realize hydrophobicity. However, if ashes accumulate on the surface of a material, the EM wave action should also be studied. Furthermore, natural flow field is ordinarily not neutral, containing loads of soluble components. Thus, salt‐alkali resistance is also a research spot for the future.

## 10 Conclusion and Outlook

Stimulated by swiftly growing electronic devices and communication techniques, more researchers have devoted themselves to the research and development of EMAS materials ever since the early 2000s.^[^ ^107^ ^]^ Traditional metal powders and coatings have been widely synthesized,^[^ ^108^ ^]^ exhibiting outstanding absorbing or shielding properties, along with various microstructure designs, including MOFs, microspheres, 2D sheets, nanowires, and nanofibers.^[^ ^109^ ^]^ However, as the researchers go deeper, it has been found that the mere EMAS ability is not enough for practical application, which requires multifunctions.^[^ ^110^ ^]^ Under these circumstances, a shift has come into being from traditional chemical synthesis to macroscopic EM device design.^[^ ^111^ ^]^

In a word, focusing on development requirements, this review summarized commonly occurring field‐responsive materials from the perspective of field theory in physics. Several external fields were concluded, including temperature, light, space–time, electrical, wind, density, and flow fields. Synthesis methods, micro‐ or macrostructures, and EMAS properties were discussed as well.

First, as shown in **Table** **1**, the most widely discussed shielding materials are graphene, Ag, MXene, and CNTs, and each one of them can respond to at least one external field. Most of the earlier material systems are mainly focused on temperature, light, and space–time fields, because of the high electrical and thermal conductivity. By construction of abundant internal electron conductive networks, high‐EMI SE can be realized with a low filling ratio of shielding materials, thus leading to high visible light transparency. Researchers should concentrate on electrical‐ or wind field‐responsive shielding materials’ design in terms of the current condition. Considering macroformations of graphene, Ag, MXene, and CNTs are mainly films, and aerogels, patches, and wearable devices should also be developing focal points.

**Table 1:** The field effects of common shielding materials

Materials	Temperature	Light	Space–time	Electrical	Wind	Density	Flow	Ref.
Graphene	√	√	√	×	×	×	×	34/35/36
Ag	√	√	×	×	×	×	√	28/34/55/57
MXene	×	√	×	×	×	×	×	58
CNTs	√	×	√	×	√	×	×	76/77/89
SiOC–ZrB_2_	×	×	×	×	√	×	×	100

Second, **Table** **2** shows that commonly applied absorbing materials are MXene/GO, polymers, SiC, ITO, and CNTs, focusing on temperature, space–time, wind, and flow fields. By the structure of foams and gels, thermal conduction or isolation is easily to accomplish. 3D printing is also proved as an efficient method to achieve a complicated macrostructure or multilayered coatings. Compared with shielding materials, absorbing materials are much more multifunctional. Still, there is development space in developing electrical‐ or light‐responsive absorbers, especially the absorbing visible transparent coatings. Transparent absorbing coatings are of significant value to EM windows, which is going to be a hotspot in the future.

**Table 2:** The field effects of common absorbing materials

Materials	Temperature	Light	Space–time	Electrical	Wind	Density	Flow	Ref.
MXene/GO	√	×	×	×	√	√	√	27/45/90
Cellulose/chitosan/polyaniline	√	×	×	×	×	×	×	46
SiC	√	×	×	×	√	√	√	26/47/91/104
ITO	×	√	×	×	×	×	×	56
CNT	×	×	√	×	×	×	×	65/75
Sn@C	×	×	×	√	×	×	×	25/85
Cement	×	×	×	×	√	×	×	98/99
Polyurethane/Fe_3_O_4_/MoS_2_	×	×	√	×	×	×	×	66

Finally, although various EMAS materials have been successfully obtained to date, the existing field‐responsive materials do not achieve excellent performance under every field. For example, flow field may erode the surface of EMAS materials, causing decrease in absorbing and shielding properties. High stability should be realized in future. Furthermore, as mentioned earlier, the current research focus should be on the exploration of new external fields, like magnetic field, stress field, and so on. It is also urgent to enrich the existing function of material system. The electrical field, density field, and light field are yet to be discovered with potential.

## Conflict of Interest

The authors declare no conflict of interest.

## References

[1] a) G. Sun , B. Dong , M. Cao , B. Wei , C. Hu , Chem. Mater. 2011, 23, 1587;

[2] a) G. Wang , Z. Gao , S. Tang , C. Chen , F. Duan , S. Zhao , S. Lin , Y. Feng , L. Zhou , Y. Qin , Acs Nano 2012, 6, 11009. 23171130 10.1021/nn304630h

[3] Z. Wang , B. Mao , Q. Wang , J. Yu , J. Dai , R. Song , Z. Pu , D. He , Z. Wu , S. Mu , Small 2018, 14, 1704332. 10.1002/smll.201704332 29665217

[4] a) Y. Zhang , W. Liu , B. Quan , G. Ji , J. Ma , D. Li , W. Meng , J. Colloid Interface Sci. 2017, 508, 462; 28858656 10.1016/j.jcis.2017.08.074

[5] a) Y. Cheng , G. Ji , Z. Li , H. Lv , W. Liu , Y. Zhao , J. Cao , Y. Du , J. Alloys Compd. 2017, 704, 289;

[6] H. Lv , Z. Yang , H. Xu , L. Wang , R. Wu , Adv. Funct. Mater. 2019, 30, 1907251.

[7] B. Zhao , G. Shao , B. Fan , W. Zhao , Y. Xie , R. Zhang , J. Mater. Chem. A 2015, 3, 10345.

[8] a) Y. Cheng , Y. Zhao , H. Zhao , H. Lv , X. Qi , J. Cao , G. Ji , Y. Du , Chem. Eng. J. 2019, 372, 390;

[9] H. Lv , G. Ji , H. Zhang , M. Li , Z. Zuo , Y. Zhao , B. Zhang , D. Tang , Y. Du , Sci. Rep. 2015, 5, 18249. 26659124 10.1038/srep18249 PMC4676003

[10] L. Yang , H. Lv , M. Li , Y. Zhang , J. Liu , Z. Yang , Chem. Eng. J. 2020, 392, 123666.

[11] a) F. Shahzad , M. Alhabeb , C. B. Hatter , B. Anasori , S. M. Hong , C. M. Koo , Y. Gogotsi , Science 2016, 353, 1137; 27609888 10.1126/science.aag2421

[12] M. Han , C. E. Shuck , R. Rakhmanov , D. Parchment , B. Anasori , C. M. Koo , G. Friedman , Y. Gogotsi , ACS Nano 2020, 14, 5008. 32163265 10.1021/acsnano.0c01312

[13] T. Yun , H. Kim , A. Iqbal , Y. S. Cho , G. S. Lee , M. K. Kim , S. J. Kim , D. Kim , Y. Gogotsi , S. O. Kim , C. M. Koo , Adv. Mater. 2020, 32, 1906769. 10.1002/adma.201906769 31971302

[14] X. Li , X. Yin , C. Song , M. Han , H. Xu , W. Duan , L. Cheng , L. Zhang , Adv. Funct. Mater. 2018, 28, 1803938.

[15] L. Wang , X. Li , Q. Li , X. Yu , Y. Zhao , J. Zhang , M. Wang , R. Che , Small 2019, 15, 1900900. 10.1002/smll.201900900 30957426

[16] T. Kim , J. Y. Bae , N. Lee , H. H. Cho , Adv. Funct. Mater. 2019, 29, 1807319.

[17] C. Zhang , C. Long , S. Yin , R. G. Song , B. H. Zhang , J. W. Zhang , D. P. He , Q. Cheng , Mater. Des. 2021, 206, 109768.

[18] a) M. Rostami , M. Jafarpour , M. H. Majles Ara , J. Alloys Compd. 2021, 872, 159656;

[19] C. Zhang , W. K. Cao , L. T. Wu , J. C. Ke , Y. Jing , T. J. Cui , Q. Cheng , Appl. Phys. Lett. 2021, 118, 133502.

[20] C. Zhang , S. Yin , C. Long , B. W. Dong , D. He , Q. Cheng , Opt. Express 2021, 29, 14078. 33985133 10.1364/OE.423245

[21] H. Lv , Z. Yang , B. Liu , G. Wu , Z. Lou , B. Fei , R. Wu , Nat. Commun. 2021, 12, 834. 33547310 10.1038/s41467-021-21103-9 PMC7864982

[22] a) A. M. Nicolson , G. F. Ross , IEEE Trans. Instrum. Meas. 1970, 19, 377;

[23] a) A. Fernandez , A. Valenzuela , Electron. Lett. 1985, 21, 20;

[24] H. J. Kwon , J. Y. Shin , J. H. Oh , J. Appl. Phys. 1994, 75, 6109.

[25] Z. Ma , C.‐T. Cao , Q.‐F. Liu , J.‐B. Wang , Chin. Phys. Lett. 2012, 29, 038401.

[26] a) Y. K. Hong , C. Y. Lee , C. K. Jeong , D. E. Lee , K. Kim , J. Joo , Rev. Sci. Instrum. 2003, 74, 1098;

[27] M. C. Vu , P. J. Park , S.‐R. Bae , S. Y. Kim , Y.‐M. Kang , W. K. Choi , M. A. Islam , J. C. Won , M. Park , S.‐R. Kim , J. Mater. Chem. A 2021, 9, 8527.

[28] Y. Yang , S. Chen , W. Li , P. Li , J. Ma , B. Li , X. Zhao , Z. Ju , H. Chang , L. Xiao , H. Xu , Y. Liu , ACS Nano 2020, 14, 8754. 32538618 10.1021/acsnano.0c03337

[29] Z. Barani , F. Kargar , A. Mohammadzadeh , S. Naghibi , C. Lo , B. Rivera , A. A. Balandin , Adv. Electron. Mater. 2020, 6, 2000520.

[30] J. Li , L. Huang , Y. Yuan , Y. Li , X. He , Mat. Sci. Eng. B 2021, 263, 114893.

[31] H. Jia , Q.‐Q. Kong , X. Yang , L.‐J. Xie , G.‐H. Sun , L.‐L. Liang , J.‐P. Chen , D. Liu , Q.‐G. Guo , C.‐M. Chen , Carbon 2021, 171, 329.

[32] M. Zhou , G. Wan , P. Mou , S. Teng , S. Lin , G. Wang , J. Mater. Chem. C 2021, 9, 869.

[33] W. Liu , T. Yao , K. Jia , J. Gu , D. Wang , X. Wei , J. Mater. Sci.: Mater. Electron. 2021, 32, 4393.

[34] Q.‐W. Wang , H.‐B. Zhang , J. Liu , S. Zhao , X. Xie , L. Liu , R. Yang , N. Koratkar , Z.‐Z. Yu , Adv. Funct. Mater. 2019, 29, 1806819.

[35] S. Lu , B. Li , K. Ma , S. Wang , X. Liu , Z. Ma , L. Lin , G. Zhou , D. Zhang , Appl. Phys. A 2020, 126, 513.

[36] W. Chen , L. X. Liu , H. B. Zhang , Z. Z. Yu , ACS Nano 2021, 15, 7668. 33861590 10.1021/acsnano.1c01277

[37] J. Li , X. Zhao , W. Wu , X. Ji , Y. Lu , L. Zhang , Chem. Eng. J. 2021, 415, 129054.

[38] L. Liang , Q. Li , X. Yan , Y. Feng , Y. Wang , H. B. Zhang , X. Zhou , C. Liu , C. Shen , X. Xie , ACS Nano 2021, 15, 6622. 33780231 10.1021/acsnano.0c09982

[39] Z. Zhang , J. Tan , W. Gu , H. Zhao , J. Zheng , B. Zhang , G. Ji , Chem. Eng. J. 2020, 395, 125190.

[40] C. Liang , Z. Wang , Chem. Eng. J. 2019, 373, 598.

[41] W. Gu , J. Sheng , Q. Huang , G. Wang , J. Chen , G. Ji , Nano-Micro Lett. 2021, 13, 102. 10.1007/s40820-021-00635-1 PMC8021664 34138342

[42] H. Zhao , Y. Cheng , Z. Zhang , B. Zhang , C. Pei , F. Fan , G. Ji , Carbon 2021, 173, 501.

[43] Y. Li , X. Liu , X. Nie , W. Yang , Y. Wang , R. Yu , J. Shui , Adv. Funct. Mater. 2019, 29, 1807624.

[44] W. Gu , J. Tan , J. Chen , Z. Zhang , Y. Zhao , J. Yu , G. Ji , ACS Appl. Mater. Interfaces 2020, 12, 28727. 32479045 10.1021/acsami.0c09202

[45] Z. Deng , P. Tang , X. Wu , H. B. Zhang , Z. Z. Yu , ACS Appl. Mater. Interfaces 2021, 13, 20539. 33877797 10.1021/acsami.1c02059

[46] H. Yu , Z. Tong , B. Zhang , Z. Chen , X. Li , D. Su , H. Ji , Chem. Eng. J. 2021, 418, 129342.

[47] A. Choe , J. Yeom , Y. Kwon , Y. Lee , Y.‐E. Shin , J. Kim , H. Ko , Mater. Horiz. 2020, 7, 3258.

[48] C. Jiang , D. Tan , Q. Li , J. Huang , J. Bu , L. Zang , R. Ji , S. Bi , Q. Guo , ACS Appl. Mater. Interfaces 2021, 13, 15525. 33769027 10.1021/acsami.1c00590

[49] G. Deng , K. Lv , H. Sun , J. Yang , Z. Yin , B. Chi , X. Li , J. Phys. D: Appl. Phys. 2021, 54, 165301.

[50] X. Zhu , A. Guo , Z. Yan , F. Qin , J. Xu , Y. Ji , C. Kan , Nanoscale 2021, 13, 8067. 33881446 10.1039/d1nr00977j

[51] C. Ma , W.‐T. Cao , W. Zhang , M.‐G. Ma , W.‐M. Sun , J. Zhang , F. Chen , Chem. Eng. J. 2021, 403, 126438.

[52] Q. Xie , Z. Yan , S. Wang , Y. Wang , L. Mei , F. Qin , R. Jiang , Adv. Eng. Mater. 2021, 23, 2100283.

[53] A. S. Voronin , Y. V. Fadeev , I. V. Govorun , I. V. Podshivalov , M. M. Simunin , I. A. Tambasov , D. V. Karpova , T. E. Smolyarova , A. V. Lukyanenko , A. A. Karacharov , I. V. Nemtsev , S. V. Khartov , J. Mater. Sci. 2021, 56, 14741.

[54] Y. Zhang , H. Dong , N. Mou , H. Li , X. Yao , L. Zhang , Nanoscale 2021, 13, 7831. 33876797 10.1039/d0nr08640a

[55] J. Xu , Y. Fan , X. Su , J. Guo , J. Zhu , Q. Fu , F. Zhang , Opt. Mater. 2021, 113, 110852.

[56] Z. Gao , Q. Fan , X. Tian , C. Xu , Z. Meng , S. Huang , T. Xiao , C. Tian , Opt. Mater. 2021, 112, 110793.

[57] C. Yang , S. Niu , H. Chang , Y. Wang , Y. Feng , Y. Zhang , G. Li , S. Chen , Y. Qu , L. Xiao , Opt. Express 2021, 29, 13610. 33985093 10.1364/OE.424226

[58] L. C. Wang , W. L. Song , Y. J. Zhang , M. J. Qu , Z. Zhao , M. Chen , Y. Yang , H. Chen , D. Fang , Adv. Funct. Mater. 2020, 30, 1909087.

[59] X. Li , Y. Zhu , X. Liu , B. Bin Xu , Q. Ni , Compos. Struct. 2020, 238, 111954.

[60] A. V. Menon , G. Madras , S. Bose , ACS Appl. Polym. Mater. 2019, 1, 2417.

[61] H. Wei , X. Cauchy , I. O. Navas , Y. Abderrafai , K. Chizari , U. Sundararaj , Y. Liu , J. Leng , D. Therriault , ACS Appl. Mater. Interfaces 2019, 11, 24523. 31187627 10.1021/acsami.9b04245

[62] Y. Yan , H. Xia , Y. Qiu , Z. Xu , Q.‐Q. Ni , RSC Adv. 2019, 9, 9401. 35520719 10.1039/c9ra00028c PMC9062159

[63] D. Kong , J. Li , A. Guo , X. Xiao , Chem. Eng. J. 2021, 408, 127365.

[64] H. Lu , Z. Li , X. Qi , L. Xu , Z. Chi , D. Duan , M. D. Z. Islam , W. Wang , X. Jin , Y. Zhu , Y. Fu , L. Cui , Y. Zhuang , Y. Dong , Compos. Sci. Technol. 2021, 207, 108697.

[65] J. Lu , Y. Zhang , Y. Tao , B. Wang , W. Cheng , G. Jie , L. Song , Y. Hu , J. Colloid Interface Sci. 2021, 588, 164. 33388582 10.1016/j.jcis.2020.12.076

[66] A. V. Menon , G. Madras , S. Bose , Chem. Eng. J. 2018, 352, 590.

[67] X. Jia , B. Shen , L. Zhang , W. Zheng , Chem. Eng. J. 2021, 405, 126927.

[68] S. Zhu , Q. Zhou , M. Wang , J. Dale , Z. Qiang , Y. Fan , M. Zhu , C. Ye , Compos., Part B 2021, 204, 108497.

[69] M. Zhou , Q. Yan , Q. Fu , H. Fu , Carbon 2020, 169, 235.

[70] A. V. Menon , G. Madras , S. Bose , Chem. Eng. J. 2019, 366, 72.

[71] L. Chen , K. Guo , S.‐L. Zeng , L. Xu , C.‐Y. Xing , S. Zhang , B.‐J. Li , Carbon 2020, 162, 445.

[72] E. S. Ko , C. Kim , Y. Choi , K. Y. Lee , Carbohydr. Polym. 2020, 245, 116496. 32718609 10.1016/j.carbpol.2020.116496

[73] H. J. Sim , D. W. Lee , H. Kim , Y. Jang , G. M. Spinks , S. Gambhir , D. L. Officer , G. G. Wallace , S. J. Kim , Carbon 2019, 155, 499.

[74] Y. M. Wang , M. Pan , X. Y. Liang , B. J. Li , S. Zhang , Macromol. Rapid Commun. 2017, 38, 1700447. 10.1002/marc.201700447 29095546

[75] Y. Zhu , J. Liu , T. Guo , J. J. Wang , X. Tang , V. Nicolosi , ACS Nano 2021, 15, 1465. 33397098 10.1021/acsnano.0c08830

[76] Y. Zhan , Y. Cheng , N. Yan , Y. Li , Y. Meng , C. Zhang , Z. Chen , H. Xia , Chem. Eng. J. 2021, 417, 129339.

[77] B. Lou , A. Sha , D. M. Barbieri , Z. Liu , F. Zhang , W. Jiang , Mater. Struct. 2021, 54, 9.

[78] C. Ma , W. Wang , Q. Wang , N. Sun , S. Hu , S. Wei , H. Feng , X. Hao , W. Li , D. Kong , S. Wang , S. Chen , J. Colloid Interface Sci. 2021, 594, 604. 33780765 10.1016/j.jcis.2021.03.048

[79] H. Lv , Z. Yang , S. J. H. Ong , C. Wei , H. Liao , S. Xi , Y. Du , G. Ji , Z. J. Xu , Adv. Funct. Mater. 2019, 29, 1900163.

[80] H. Lv , Z. Yang , P. L. Wang , G. Ji , J. Song , L. Zheng , H. Zeng , Z. J. Xu , Adv. Mater. 2018, 30, 1706343. 10.1002/adma.201706343 29512210

[81] L. Liu , L. B. Kong , S. Matitsine , Appl. Phys. Lett. 2008, 93, 113106.

[82] L. Liu , Z. H. Yang , L. B. Kong , W. Y. Yin , S. Wang , Appl. Phys. A 2012, 108, 843.

[83] X. Chen , Z. Tian , Y. Lu , Y. Xu , X. Zhang , C. Ouyang , J. Gu , J. Han , W. Zhang , Adv. Opt. Mater. 2019, 8, 1900660.

[84] Z. Cai , L. Su , H. Wang , M. Niu , L. Tao , D. Lu , L. Xu , M. Li , H. Gao , ACS Appl. Mater. Interfaces 2021, 13, 16704. 33797879 10.1021/acsami.1c02906

[85] Y. Y. Wang , Z. H. Zhou , C. G. Zhou , W. J. Sun , J. F. Gao , K. Dai , D. X. Yan , Z. M. Li , ACS Appl. Mater. Interfaces 2020, 12, 8704. 31971778 10.1021/acsami.9b21048

[86] Z. Wang , R. Wei , J. Gu , H. Liu , C. Liu , C. Luo , J. Kong , Q. Shao , N. Wang , Z. Guo , X. Liu , Carbon 2018, 139, 1126.

[87] K. Su , Y. Wang , K. Hu , X. Fang , J. Yao , Q. Li , J. Yang , ACS Appl. Mater. Interfaces 2021, 13, 22017. 33909396 10.1021/acsami.1c03543

[88] S. Li , J. Wang , Z. Zhu , D. Liu , W. Li , G. Sui , C. B. Park , J. Mater. Chem. A 2021, 9, 358.

[89] Y. Zhang , J. Yu , J. Lu , C. Zhu , D. Qi , J. Alloys Compd. 2021, 870, 159442.

[90] L. Li , Y. Cao , X. Liu , J. Wang , Y. Yang , W. Wang , ACS Appl. Mater. Interfaces 2020, 12, 27350. 32437119 10.1021/acsami.0c05692

[91] Y. Yuan , Y. Feng , X. Zhao , M. Yang , H. Lu , J. Li , Y. Du , Y. Li , X. He , Ceram. Int. 2018, 44, 6673.

[92] C. Liang , Y. Du , Y. Wang , A. Ma , S. Huang , Z. Ma , Adv. Compos. Hybrid Mater., 10.1007/s42114-021-00274-5.

[93] Z. Qu , K. Wang , C.‐A. Xu , Y. Li , E. Jiao , B. Chen , H. Meng , X. Cui , J. Shi , K. Wu , Chem. Eng. J. 2021, 421, 129729.

[94] H. Guo , Z. Wang , D. An , J. Huo , J. Cleaner Prod. 2021, 283, 124614.

[95] G. Ma , Y. Zhang , X. Liu , Constr. Build. Mater. 2020, 265, 120771.

[96] Y. Jia , T. D. Ajayi , M. A. Roberts, Jr. , C. C. Chung , C. Xu , ACS Appl. Mater. Interfaces 2020, 12, 46254. 32965104 10.1021/acsami.0c08479

[97] X. Li , G. Zhou , Q. Yang , X. Zhu , G. Ren , L. Liu , Ceram. Int. 2020, 46, 2294.

[98] F. Ye , Q. Song , Z. Zhang , W. Li , S. Zhang , X. Yin , Y. Zhou , H. Tao , Y. Liu , L. Cheng , L. Zhang , H. Li , Adv. Funct. Mater. 2018, 28, 1707205.

[99] D. Gu , X. Shi , R. Poprawe , D. L. Bourell , R. Setchi , J. Zhu , Science 2021, 372, 932. 10.1126/science.abg1487 34045326

[100] Y. Zuo , X. Su , X. Li , Z. Yao , T. Yu , J. Zhou , J. Li , J. Lu , J. Ding , Carbon 2020, 167, 62.

[101] S. Singh , A. Sinha , R. H. Zunke , A. Kumar , D. Singh , Adv. Powder Technol. 2018, 29, 2019.

[102] N. Rezazadeh , A. Kianvash , P. Palmeh , J. Appl. Polym. Sci. 2018, 135, 46565.

[103] M. Zhang , C. Han , W. Q. Cao , M. S. Cao , H. J. Yang , J. Yuan , Nano-Micro Lett. 2021, 13, 27. 10.1007/s40820-020-00552-9 PMC8187527 34138252

[104] Z. Cai , L. Su , H. Wang , M. Niu , H. Gao , D. Lu , M. Li , ACS Appl. Mater. Interfaces 2020, 12, 8555. 31985205 10.1021/acsami.9b20636

[105] T.‐T. Li , Y. Wang , H.‐K. Peng , X. Zhang , B.‐C. Shiu , J.‐H. Lin , C.‐W. Lou , Compos., Part A 2020, 128, 105685.

[106] L. Huang , Y. Duan , X. Dai , Y. Zeng , G. Ma , Y. Liu , S. Gao , W. Zhang , Small 2019, 15, 1902730. 10.1002/smll.201902730 31402564

[107] a) J. Wang , Z. Jia , X. Liu , J. Dou , B. Xu , B. Wang , G. Wu , Nano-Micro Lett. 2021, 13, 175; 10.1007/s40820-021-00704-5 PMC8368508 34398334

[108] X. Zhang , Z. C. Liu , B. W. Deng , L. Cai , Y. Y. Dong , X. J. Zhu , W. Lu , Chem. Eng. J. 2021, 419, 129547.

[109] Y. L. Wang , S. H. Yang , H. Y. Wang , G. S. Wang , X. B. Sun , P. G. Yin , Carbon 2020, 167, 485.

[110] a) S. Gao , G. S. Wang , L. Guo , S. H. Yu , Small 2020, 16, 1906668; 10.1002/smll.201906668 32297713

[111] a) Y. F. Pan , G. S. Wang , L. Liu , L. Guo , S. H. Yu , Nano Res. 2017, 10, 284;

[112] X. Shi , D. Gu , Y. Li , D. Dai , Q. Ge , Y. Sun , H. Chen , Opt. Laser Technol. 2021, 138, 106917.

[113] C. Chen , Y. Zhao , W. Wei , J. Tao , G. Lei , D. Jia , M. Wan , S. Li , S. Ji , C. Ye , J. Mater. Chem. C 2017, 5, 2240.

